# Stepwise virus assembly in the cell nucleus revealed by spatiotemporal click chemistry of DNA replication

**DOI:** 10.1126/sciadv.adq7483

**Published:** 2024-10-25

**Authors:** Alfonso Gomez-Gonzalez, Patricia Burkhardt, Michael Bauer, Maarit Suomalainen, José María Mateos, Morten O. Loehr, Nathan W. Luedtke, Urs F. Greber

**Affiliations:** ^1^ Department of Molecular Life Sciences, University of Zurich (UZH), Zurich, Switzerland.; ^2^ Center for Microscopy and Image Analyses, University of Zurich (UZH), Zurich, Switzerland.; ^3^ Department of Chemistry, McGill University, Montréal, QC, Canada.

## Abstract

Biomolecular assemblies are fundamental to life and viral disease. The spatiotemporal coordination of viral replication and assembly is largely unknown. Here, we developed a dual-color click chemistry procedure for imaging adenovirus DNA (vDNA) replication in the cell nucleus. Late- but not early-replicated vDNA was packaged into virions. Early-replicated vDNA segregated from the viral replication compartment (VRC). Single object tracking, superresolution microscopy, fluorescence recovery after photobleaching, and correlative light-electron microscopy revealed a stepwise assembly program involving vDNA and capsid intermediates. Depending on replication and the scaffolding protein 52K, late-replicated vDNA with rapidly exchanging green fluorescent protein–tagged capsid linchpin protein V and incomplete virions emerged from the VRC periphery. These nanogel-like puncta exhibited restricted movements and were located with the capsid proteins hexon, VI, and virions in the nuclear periphery, suggestive of sites for virion formation. Our findings identify VRC dynamics and assembly intermediates, essential for stepwise productive adenovirus morphogenesis.

## Teaser

Nanogel-like viral assembly intermediates emerge as bubbles from the nuclear replication compartment and give rise to virions.

## INTRODUCTION

Eukaryotic cells evolved a membrane-enclosed nuclear compartment to maintain genetic information and regulate DNA activity through diverse processes, including transcription, replication, editing, and repair (*1*). The nucleus is highly dynamic not only in cell division, apoptosis, spermatogenesis, and erythropoiesis but also in interphase (*2*–*5*). Chromatin dynamics involves assembly, condensation, and decondensation processes and has been explored through high-speed superresolution fluorescence microscopy (*6*, *7*), cryogenic electron tomography (*8*, *9*), and increasingly deep learning and artificial neural networks in computer vision procedures (*10*–*12*). These approaches have provided crucial in situ information on transcription initiation, elongation and processing (*13*), activation of origins in DNA replication (*14*), chemical modifications of DNA and associated histones (*15*), DNA repair (*16*), or the breakdown and reformation of the nuclear envelope in mitosis (*17*).

Further to processes in growth and homeostasis, the nucleus has been subject to subversion by a range of viruses, including DNA viruses, retroviruses, and negative-strand RNA viruses, some of them causing severe human and societal ailments, including cancer and pandemics (*18*–*20*). For example, DNA tumor viruses, including polyoma, papilloma, herpes, or adenovirus (AdV) disrupt homeostasis by competing with the host for nucleotides and blocking the synthesis of cellular DNA while promoting viral DNA (vDNA) replication in so-called viral replication compartments (VRCs), where viral and host proteins mediate vDNA replication, packaging, and virion assembly (*21*–*26*).

Here, we address how human AdV exploits a de novo subnuclear compartment for virion assembly. AdVs are non-enveloped double-strand DNA viruses, prevalent in humans and other vertebrates, and their vectors are widely used in both preclinical and clinical gene therapies (*27*–*29*). They comprise more than 100 natural human types, based on serology and genome sequence data (http://hadvwg.gmu.edu/). AdVs infect the respiratory organs, eyes, kidneys, and gastrointestinal tract with self-limiting disease and dissemination to immune cells (*30*, *31*). They deliver a proteinaceous capsid into the cytosol with vDNA condensed and bundled by viral proteins VII, X, and V (*32*–*35*), engage microtubule-dependent motors for nuclear targeting, dock to the nuclear pore complex (*36*), release the DNA-capsid linchpin protein V (*32*, *33*), uncoat the vDNA (*37*), and deliver the genome into the nucleus, indicated by single-genome tracking with copper(I) azide-alkyne cycloaddition (CuAAC) click chemistry (*21*, *33*, *38*).

The AdV genome contains eight transcription units, each with its own promoter [reviewed in (*39*, *40*)]. Expression of early viral genes drives the cell cycle, blocks apoptosis, tunes metabolism, and antagonizes the host cell immune response in preparation for vDNA replication (*41*). Besides cellular factors such as NF1, three viral proteins directly promote the formation of the VRC, DNA polymerase (Pol), terminal protein (TP) serving as a polymerase primer on the 5′ ends of the linear vDNA, and single-strand DNA binding protein (DBP; also known as 72K), where DBP might contribute by forming a gel-like substance (*42*, *43*). VRCs grow radially throughout infection covering a large fraction of the nuclear volume (*44*), and up to 30,000 progeny virions each with a genome of about 36 kbp (*45*). VRCs produce an excess of genomes over progeny particles.

Upon intermediate and late viral gene expression, virus particles are assembled. Intermediate-expressed proteins mediate vDNA packaging (IVa2, 22K, 33K, and IIIa) and scaffolding of nascent particles (52K) (*46*). Proteins from a differentially spliced and polyadenylated primary transcript of the major late (ML) transcription units (L1 to L5 mRNAs) comprise the capsid proteins hexon, penton base, IIIa, fiber, VI, VIII, IX, L3-p23/protease, and the vDNA-associated proteins IVa2, V, VII, X, and TP (*40*). Virus particles are proteolytically processed into infectious mature virions (*47*) and released from the cell by yet-unknown mechanisms (*40*, *48*).

Despite detailed knowledge of the viral life cycle from more than 70 years of adenovirology, it is not yet resolved how AdV particles are formed. There have been two competing models. The long-standing sequential assembly model postulates that vDNA is encapsidated into preformed empty particles, largely based on observations that AdV-C5 virions bear a packaging protein (IVa2) that binds adenosine 5′-triphosphate (ATP) and produces empty particles (*49*–*51*). The sequential model has been adapted from bacteriophages, where genome insertion into preformed capsids is firmly established by genetic and biochemical evidence including ATP-dependent packaging motors and stable intermediates captured upon slowing the assembly reactions in vitro [reviewed in (*52*, *53*)]. An alternative view has originally been proposed for human AdV-B3 based on the isolation of two types of DNA-less incomplete particles, one with a single discontinuity and the other one with a more amorphous structure of hexon aggregates, the formation of which depended on ongoing protein synthesis (*54*). More recent EM experiments have provided in situ evidence for semi-closed capsid assembly intermediates suggesting that capsid assembly and vDNA encapsidation are concurrent processes (*55*).

Our results support the concurrent model and identify distinct steps in AdV morphogenesis. Time-resolved dual-color pulse-chase click chemistry visualizes replicating AdV-C5 DNA, and live-cell analyses of the green fluorescent protein (GFP)–V capsid-vDNA linchpin protein reveal assembly intermediates (*32*, *33*). Together with single-particle tracking, superresolution microscopy, fluorescence recovery after photobleaching (FRAP), and correlative light-electron microscopy (CLEM) data, these data show the segregation (bubbling) of newly replicated vDNA from the VRC, restricted diffusion of nanogel-like puncta containing GFP-V and vDNA in the 52K compartment around the VRC, and virion formation from nanogel-like puncta preferentially in the nuclear periphery.

## RESULTS

### Vinyl-modified nucleosides are incorporated into AdV-C5 VRC and virions and tag genomes in virus entry

**Fig. 1.**
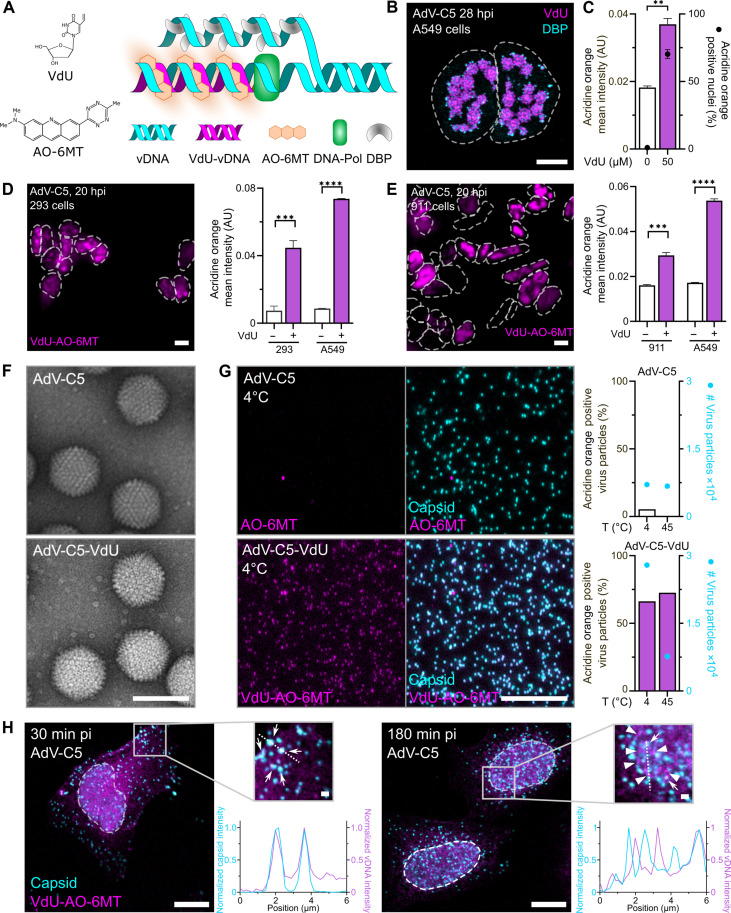
Incorporation of VdU into AdV-infected A549 cells and E1-expressing helper cell lines and virions isolated from these cells. (**A**) Tagging of newly synthesized VdU-modified vDNA with acridine orange–coupled 6-methyl-tetrazine (AO-6MT). (**B**) VdU incorporation during AdV-C5 replication. A549 cells were inoculated with AdV [multiplicity of infection (MOI) of 3] at 37°C for 60 min, washed, and fixed at 28 hpi. VdU was added 4 hours before fixation. Samples were immuno-stained for DBP and clicked with AO-6MT. Scale bar, 10 μm. (**C**) VdU incorporation into A549 cells infected with AdV-C5 (MOI of 1.5). Nuclei were segmented on the basis of 4′,6-diamidino-2-phenylindole (DAPI) nuclear signal, and the AO signal was quantitated in the nuclear area. Data represent means ± SD. Statistical significance was determined by unpaired *t* test. **P < 0.002. AU, arbitrary units. (**D** and **E**) VdU incorporation into AdV-C5–infected (MOI of 1.5) human embryonic kidney (HEK) 293 and human embryonic retina (HER) 911 cells, fixed at 20 hpi. For noninfected cells, VdU was present throughout 20 hours, and for AdV-C5–infected cells, 4 hours before fixation. Dashed lines indicate nuclear outlines. Scale bars, 10 μm. Bar graphs show quantitative analyses of VdU incorporation into HEK293 and HER911 cells. Data represent means ± SD of the AO-6MT signal over the DAPI-stained nuclei. Statistical significance was determined by nonparametric analysis of variance (ANOVA) with Holm-Sidak for multiple comparisons. ***P < 0.0002; ****P < 0.0001. (**F**) Electron micrographs of AdV-C5 and AdV-C5-VdU particles. Scale bar, 100 nm. (**G**) AdV-C5 and AdV-C5-VdU particles stained with AO-6MT. Virions were incubated at 4° or 45°C, bound to coverslips, fixed, immuno-stained for hexon, and clicked with AO-6MT, and the percentage of AO-6MT–positive virus particles was displayed. Scale bar, 10 μm. (**H**) Tracking of incoming VdU-labeled virions. Arrows indicate VdU-AO-6MT–labeled vDNA-containing viral particles; arrowheads highlight released VdU-AO-6MT vDNA. Data represent normalized intensities of capsid and vDNA across the dotted lines. Scale bar, 10 μm. Scale bar in the zoom-in, 1 μm.

Bioorthogonal click chemistry (clicking) allows biosynthetic tagging of proteins, lipids, or nucleic acids with a wide range of functionalized molecules, including fluorophores (*56*). Unlike protein labeling, DNA labeling has been limited to just a few click reactions in cells, including strain-promoted azide-alkyne cycloaddition and inverse electron demand Diels-Alder (IEDDA) (*57*). We tested if vinyl-2′-deoxy-nucleosides were incorporated into vDNA during replication using conventional IEDDA bioorthogonal labeling by substituting the bulky trans-cyclo-octane used previously with a small vinyl-tag present in 5-vinyl-2′-deoxyuridine (VdU) (*58*, *59*). VdU reacts with acridine orange–coupled 6-methyl-tetrazine (AO-6MT) (Fig. 1A). AO-6MT is a cell-permeable, double-strand DNA intercalator, which increases the IEDDA reaction rate on DNA 60,000-fold compared to nontemplated reactions (*59*).

Human lung epithelial A549 cells were infected with AdV at high multiplicity of infection (MOI of 1.5) for 24 hours, incubated with VdU for 4 hours, fixed, labeled with AO-6MT, and stained for the VRC with DBP antibodies. VdU was incorporated into replicating vDNA (DBP-positive) in a concentration-dependent manner yielding flower-shaped patterns characteristic of VRC (Fig. 1, B and C), akin to 5-ethynyl-2′-deoxycytidine (EdC)–tagged and CuAAC-stained vDNA (*21*). Comparison of different fluorescently labeled tetrazines showed that AO-6MT was superior to XFD488-6MT and 5-TAMRA-6MT, which did not react with VdU (fig. S1, A and B). Likewise, VdU was superior to 5-vinyl-2′-deoxycytidine (VdC) and 7-deaza-7-vinyl-2′-deoxyadenosine in AdV-infected cells (fig. S1C). Notably, VdU unlike EdC was incorporated into vDNA of E1-expressing human embryonic kidney (HEK) 293 and human embryonic retina (HER) 911 cells infected with AdV-C5 (Fig. 1, D and E, and fig. S1, D and E). VdU was detected in CsCl-purified AdV-C5 and E1-deleted AdV-C5 particles (AdV-C5_ΔE1-VdU) by AO-6MT labeling of virions, notably independent of capsid disruption suggesting that AO-6MT penetrates into the particles (Fig. 1, F and G, and fig. S1, F and G). VdU-tagged AdV-C5 particles could be tracked in cell entry as indicated by immuno-stained capsids colocalizing with AO-6MT click-labeled vDNA 30 min postinfection (pi), and vDNA dissociated from capsid 180 min pi (Fig. 1H). Notably, AdV-C5 progeny formation was possible in the presence of VdU (up to 50 μM; fig. S1H). Together, the data show that VdU is readily incorporated into AdV-C5 genomes and can be used to track AdV DNA dynamics, complementing EdC.

### Dual pulse-chase tagging with EdC and VdU reveals distinct functions of early- and late-replicated vDNAs

**Fig. 2.**
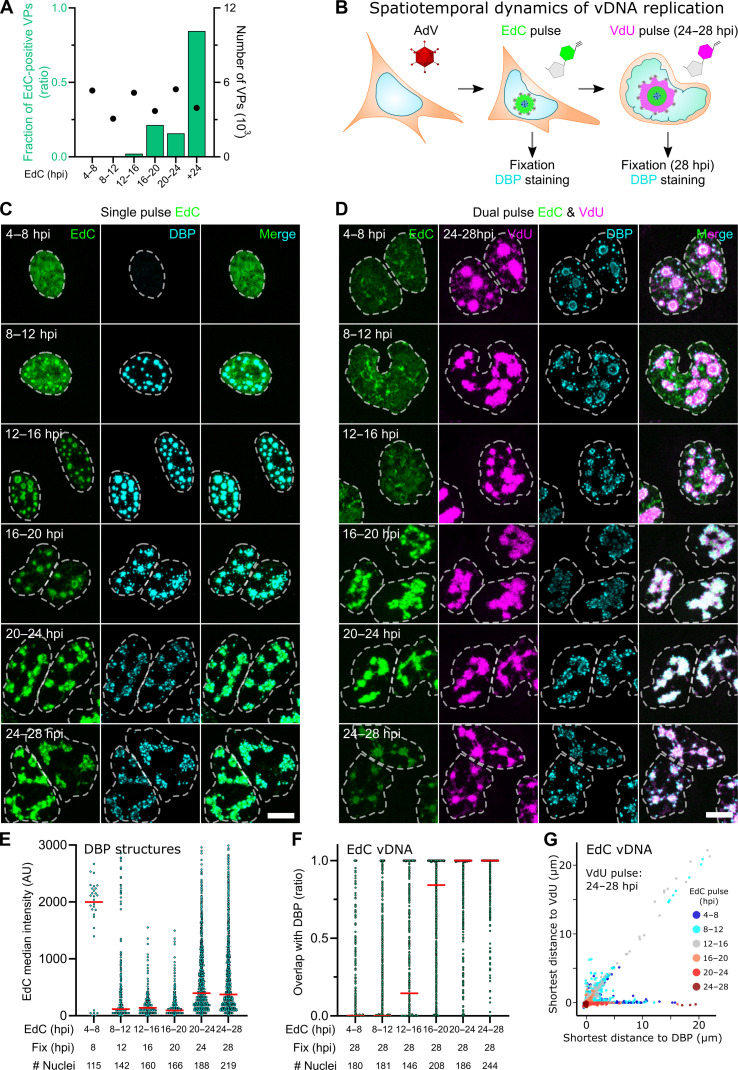
Dual-label click chemistry reveals distinct spatiotemporal features of vDNA in AdV infection. (**A**) Encapsidation of EdC into AdV particles. Cesium chloride–purified progeny virions from infected EdC-pulsed A549 cells were heat-disrupted, bound to poly-l-lysine–coated coverslips, fixed, and stained with hexon 9C12 antibody and N_3_-AlexaFluor488. Data represent the relative amount of AlexaFluor488-positive virus particles to AdV-EdC control virus. (**B**) Schematic depiction of biorthogonal dual-label pulse-chase experiment of vDNA replication. A549 cells were infected with AdV-C5 (MOI of 3) for 60 min, washed, pulse labeled with 2.5 μM EdC for 4 hours at different times pi, washed, and either fixed or tagged with 50 μM VdU 24 to28 hpi. Samples were stained with anti-DBP antibodies (cyan), clicked with AO-6MT (magenta), and clicked with N_3_-AlexaFluor647 (green). (**C**) AdV replication compartment visualized by EdC pulse labeling. Samples were prepared as in (B). Images are maximum projections. Scale bar, 10 μm. (**D**) The VRC displays distinct patterns of early- and late-replicated vDNAs. Samples preparation as in (B). Images are maximum projections. Scale bar, 10 μm. (**E**) Quantitative analysis of the EdC content in the VRC. DBP objects from (C) were 3D segmented, and the EdC-AlexaFluor647 signal was quantitated in the DBP volume. Each data point represents a DBP object. The median is shown in red. (**F**) Quantitative analysis of vDNA dissociation from DBP late in infection. EdC-labeled vDNA objects from (D) were 3D segmented, and DBP-EdC overlap was computed and compared to the 3D-segmented DBP. Each data point represents an EdC-labeled vDNA object. The median is shown in red. (**G**) Shortest distance plots of early-replicated vDNA to late-replicated vDNA from single 3D-segmented EdC-labeled vDNA surfaces to both DBP and VdU-labeled vDNA from (D), color-coded by the EdC pulse time. EdC objects in DAPI-stained nuclei were segmented as in (F).

To explore whether VdU and EdC can be used for time-resolved pulse labeling of newly synthesized vDNA, we developed a double-label approach using EdC and VdU pulses and CuAAC and IEDDA, respectively. Pulse labeling of infected cells showed that intermediate- to late-replicated vDNA tagged with EdC was incorporated into progeny virions harvested at 48 hours pi (hpi), yielding about 20% to nearly 80% EdC-positive virions with EdC pulsing at 16 to 24 and 24 to 48 hpi, respectively (Fig. 2A). We then assessed the localization of early-replicated vDNA by pulsing infected cells with EdC at 12 to 16 hpi, followed by VdU at 24 to 28 hpi or vice versa, and staining with AO-6MT (magenta) and N_3_-AlexaFluor594 (green) at 28 hpi (fig. S2, A and B). While late-replicated localized with the VRC marker DBP, a fraction of early-replicated vDNA labeled with either EdC or VdU localized outside the VRC. The data indicate that both EdC and VdU tags are suitable for labeling early- and late-replicating vDNA.

The temporal dynamics of the VRC were further studied by 4-hour EdC pulses at different times pi. As expected, the very early EdC pulse (4 to 8 hpi) revealed a fairly homogeneous nuclear pattern lacking DBP-positive compartments consistent with cellular DNA in the S phase induced by the AdV early protein E1A (Fig. 2, B to D) (*60*). When the EdC pulse was applied later in the infection, the pattern gradually changed to discrete compartments positive for DBP at 20 hpi and exhibited a noticeable increase in the mean EdC intensity at 24 hpi, indicating that VRC exhibits high temporal dynamics (Fig. 2E and fig. S2C). Of note, at 12 to 16 hpi, about 75% of the cells had developed prominent VRCs without diffuse EdC patterns. To address the spatiotemporal dynamics of VRC, we used dual-label click experiments pulsing early-replicated vDNA with EdC for 4 hours and late-replicated vDNA with VdU followed by EdC-N_3_-AlexaFluor647 and VdU-AO-6MT staining at 28 hpi (Fig. 2D). Volumetric analysis indicated an extensive overlap between VdU and the VRC marker and vice versa (fig. S2, D and E). Further analyses showed that EdC-tagged vDNA synthesized before 16 hpi poorly overlapped with DBP at 28 hpi or with VdU pulsed 24 to 28 hpi, indicating that early-replicated vDNA segregated from late-replicated vDNA, as shown by shortest distance plots between EdC and VdU vDNA 28 hpi (Fig. 2, D, F, and G).

### Viral and cellular DNA is marginalized during AdV replication

We next used the dual-label click chemistry protocol to assess the localization of host DNA in the course of infection. Similar to early-synthesized vDNA, cellular DNA tagged with VdU before infection was distinct from the VRC tagged with EdC (fig. S3, A and B). Together with live-cell imaging of VdU-AO-6MT–prelabeled cellular DNA, these results demonstrated that vDNA synthesis is not stalled, and vDNA incorporates EdC in the presence of VdU-AO-6MT–labeled cellular DNA (fig. S3, C and D, and movie S1). The data demonstrate the clustering of preexisting cellular DNA as a consequence of AdV-C5 infection, a notion which is underscored by the observation that 4′,6-diamidino-2-phenylindole (DAPI)–stained DNA condensed in the nuclear periphery as nuclei increase in size upon infection (fig. S3E).

Last, we tested if replicating vDNA could be imaged by the cell-permeable AO-6MT. A549 cells were infected with AdV-C5 (MOI of 3), pulsed with VdU at 18 to 22 hpi, and then labeled with AO-6MT. While the AO-6MT signal in the nuclei appeared to increase over time, the overall signal remained static, suggesting stalled replication (fig. S3F and movie S2). This was confirmed by an EdC pulse at 24 to 28 hpi and labeling with N_3_-AlexaFluor647, conditions which did not yield double-positive AO-6MT/N_3_-AlexaFluor647 cells (fig. S3G). Collectively, the data show that VdU-AO-6MT labeling per se is not toxic if short pulses of AO-6MT are used. Yet, prolonged treatment with AO-6MT stalls DNA replication, likely due to the bulkiness of the covalent DNA adduct.

### Early-replicated vDNA is transcriptionally active predominantly early rather than late in infection

**Fig. 3.**
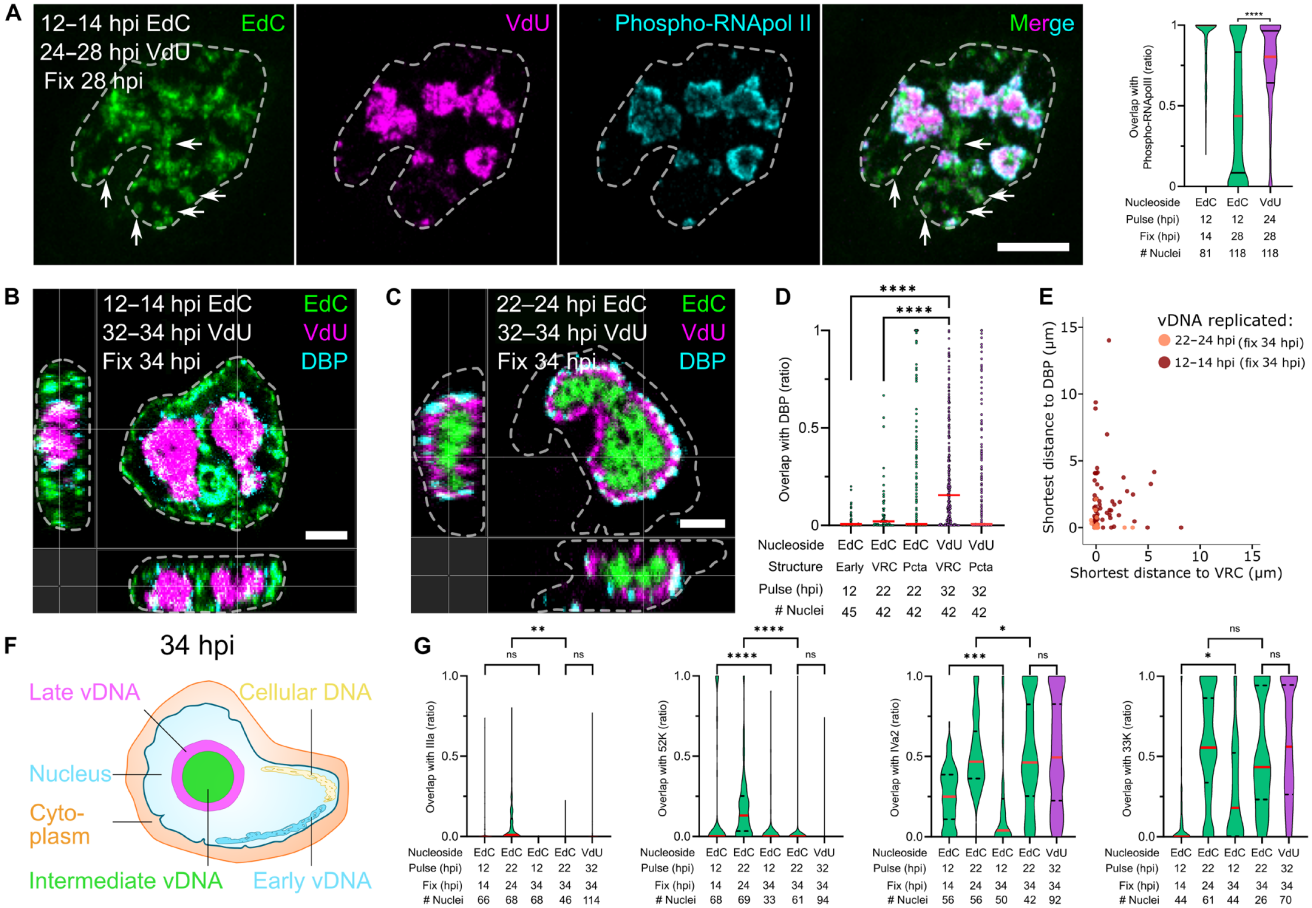
Early-replicated vDNA dissociates from VRC and becomes phospho-RNA Pol-II– and DBP-negative. (**A**) p-RNA Pol-II staining of late-replicated vDNA. Specimens were labeled with EdC (green) and VdU (magenta), fixed, and stained with anti–p-RNA Pol-II-CTD (cyan). The median of violin plotted data is shown in red. Statistical significance was determined by nonparametric ANOVA with Holm-Sidak for multiple comparisons. ****P < 0.0001. Arrows indicate p-RNA Pol-II–negative EdC regions. Images are maximum projections. Scale bar, 10 μm. (**B** and **C**) Early-replicated vDNA segregates from VRC, while intermediate-replicated vDNA constitutes the VRC center. Specimens were labeled with EdC (green) and VdU (magenta) and stained with anti-DBP (cyan) showing en face projections and orthogonal slices. Scale bars, 5 μm. (**D**) Quantitative analysis of vDNA colocalization with DBP at late stages of infection. EdC- and VdU-labeled vDNA was 3D segmented on the basis of AlexaFluor594 and AO-6MT signals. Overlap with 3D-segmented DBP objects was computed. Each data point represents a vDNA object, including puncta (Pcta). The median is shown in red. Statistical significance was determined as in (A). ****P < 0.0001. (**E**) Distance plots of vDNA objects segregated from the VRC late in infection. Data represent the shortest distance from single 3D-segmented vDNA surface objects (EdC) to the VRC (DBP and VdU). The color code indicates EdC pulse time. (**F**) Schematics depicting the spatial distribution of cellular and viral DNA late in infection. (**G**) vDNA colocalization with IIIa, 52K, IVa2, and 33K late in infection. Samples were prepared as in (B) and (C). Data distribution is shown as violin plots. The median is shown in red. Statistical significance was determined as in (A). *P < 0.03; **P < 0.0021; ***P < 0.0002; ****P < 0.0001; ns, not significant.

We next took advantage of the EdC/VdU dual-label click chemistry protocol to test whether early- and late-replicated vDNAs differed with respect to phosphorylated serine-5 RNA Pol-II (p-RNA Pol-II), which indicates transcriptional initiation (*13*). Uninfected cells showed a homogeneous p-RNA Pol-II pattern, while AdV-C5–infected cells strongly enriched active transcription sites, especially in the periphery of VRCs early in replication (12 to 14 hpi; fig. S4A). When infection progressed to 28 hpi, the early-replicated vDNA partially segregated from the late-replicated–tagged vDNA in the VRC and was p-RNA Pol-II–negative, unlike late-replicated vDNA (Fig. 3A). The loss of active p-RNA Pol-II from the early-replicated vDNA was strongly reduced by the DNA replication inhibitor 3′-deoxy-3′-fluorothymidine [DFT; (*61*)], which potently blocks AdV infection as indicated by the absence of VdU (fig. S4B). DFT also blocked the expansion of the VRC and led to the clustering of DBP, largely colocalizing with the early-replicated vDNA (fig. S4C). Together, these results show that early-replicated vDNA is transcriptionally active early in infection, segregates from the VRC, and is transcriptionally attenuated later in infection depending on vDNA replication. Notably, early-replicated vDNA is not incorporated into progeny virions (see Fig. 2A).

### Late-replicated vDNA in VRC contains vDNA packaging proteins IVa2 and 33K

We next analyzed the subnuclear localization of early-and intermediate-replicated vDNAs pulsed with EdC at 12 to 14 and 22 to 24 hpi, respectively, and compared it to the DBP-positive VRC tagged with VdU at 32 to 34 hpi. While the early-replicated vDNA was segregated from the VRC, the intermediate-replicated vDNA was enclosed by DBP and VdU-AO-6MT, as indicated by volumetric analyses (Fig. 3, B to E). The formation of AdV progeny requires vDNA replication and also the synthesis of structural proteins from alternatively spliced late (L) mRNAs (L1 to L4) involving transactivation by the intermediate proteins L4-IVa2 and L4-22K (*62*–*64*).

We used immunofluorescence microscopy to map the proteins L1-IIIa, L1-52K, L4-IVa2, and L4-33K with respect to early- and late-replicated vDNAs. It was recently reported that IIIa and 52K were homogeneously distributed around the VRC (*65*). Likewise, we observed IIIa and 52K to be homogeneously distributed around early- and late-replicated vDNAs without notable VRC overlap, although IIIa was also found in the cytoplasm (Fig. 3G and fig. S4, D to F), consistent with the notion that IIIa/52K form a condensate around the VRC (*65*). In contrast, a distinct pattern was obtained with IVa2, which cooperatively binds to the vDNA packaging sequences for virion assembly, together with IIIa, 52K, and 22K (*51*, *66*–*68*). About 25% of early-replicated vDNA overlapped with IVa2 at 14 hpi but almost none at 34 hpi (Fig. 3G and fig. S4G). Notably, about half of the late-replicated vDNA was IVa2-positive at 34 hpi, suggesting that IVa2 preferentially marks packaging active vDNA. Similar to IVa2, 33K strongly overlapped with late- and, to a lesser extent, early-replicated vDNA (Fig. 3G and fig. S4H), consistent with the notion that 33K is involved in vDNA packaging and alternative splicing of late AdV transcripts (*69*, *70*). Tentatively, these data suggest that a fraction of early-replicated vDNA may be engaged in alternative splicing, possibly of ML transcripts.

Together, the data provide evidence for functionally diverse pools of replicated vDNA. Early-replicated vDNA is phospho-RNA Pol-II–positive early, and then depending on viral replication segregates from VRC to become phospho-RNA Pol-II–negative late in infection. At the late stage of infection when virion assembly takes place, the large majority of early-replicated vDNA loses protein IVa2 as well as DBP (see Figs. 2D and 3G) while gaining an association with 33K suggesting that this pool of vDNA is no longer engaged in replication. In contrast, the late-replicated vDNA is largely positive for both IVa2 and 33K in the VRC, suggesting that this pool plays an active role in vDNA packaging.

### Punctuate objects containing intermediate- and late-replicated vDNAs are located between the VRC and the nuclear rim

**Fig. 4.**
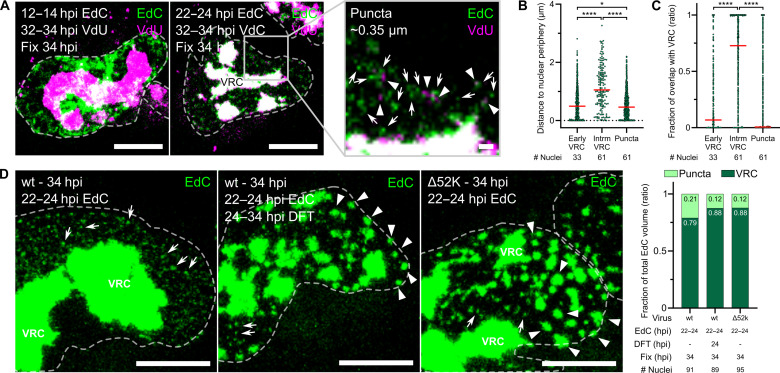
Late-replicated vDNA bubbles from VRC as distinct punctuate structures. (**A**) Samples were prepared as described in Fig. 3 (B and C). Arrows in the zoom-in indicate single EdC puncta (EdC pulse, 22 to 24 hpi); arrowheads indicate single VdU puncta (VdU pulse, 32 to 34 hpi). Images are maximum projections. Scale bar, 10 μm. Scale bar in the zoom-in, 1 μm. (**B**) Distribution profile of early VRC, intermediate VRC, and nascent vDNA puncta across the nucleus. Samples were prepared as in Fig. 3 (B and C). Shortest distance plots from 3D-segmented EdC-labeled surfaces to the nuclear rim defined by the DAPI signal. Statistical significance was determined as indicated in Fig. 3A. *P < 0.03; ****P < 0.0001. (**C**) Colocalization of early VRC, intermediate VRC, and nascent vDNA puncta with 3D-segmented VRC defined by a late VdU pulse 32 to 34 hpi. Samples prepared as described in Fig. 3 (B and C). Each data point represents one EdC-vDNA object. The median is shown in red. Statistical significance was determined as in Fig. 3A. ****P < 0.0001. (**D**) Nascent vDNA puncta depend on the 52K protein and vDNA replication. A549 cells were infected with AdV-C5 wt or Δ*L1-52K*, pulsed with 2.5 μM EdC, and treated with 2.5 μM DFT 24 to 34 hpi. Samples were clicked with N_3_-AlexaFluor594. Data represent the proportion of nascent vDNA puncta and VRC normalized to the total EdC volume. Arrows indicate small vDNA objects (puncta); arrowheads indicate large vDNA objects distinct from VRC. Images are maximum projections. Scale bars, 10 μm.

Besides prominent VRCs, the infected nuclei invariably contained distinct vDNA puncta stained with VdU-AO-6MT at 32 to 34 hpi (see red squares in fig. S4, E to H). Similar puncta were observed with intermediate-replicated vDNA pulsed with EdC at 22 to 24 hpi, many of them located within about 0.5 μm from the nuclear rim (defined by DAPI staining), as indicated by shortest distance plots (Fig. 4, A to C). All the vDNA puncta were DBP-, IVa2-, IIIa-, and 52K-negative, although ~50% of the late-replicated ones were positive for 33K (Fig. 3D and fig. S5, A to D). Notably, the EdC-positive vDNA puncta strongly depended on vDNA replication and the virus assembly protein 52K, as indicated by DFT treatment of AdV-C5–infected cells and infections with AdV-C5 pm8001 lacking *L1-52K*, respectively (Fig. 4D) (*71*). Notably, DFT did not prevent the formation of 52K condensates but led to VRC fragmentation or impaired VRC coalescence, possibly by blocking the incorporation of nucleosides into nascent vDNA (fig. S5, E and F). Together with the finding that both intermediate- and late-replicated vDNAs are packaged into virions (Fig. 2A), the results suggest that the vDNA puncta represent bona fide particle assembly intermediates.

### Puncta containing vDNA and GFP-V emerge from the VRC depending on replication and the assembly protein 52K

**Fig. 5.**
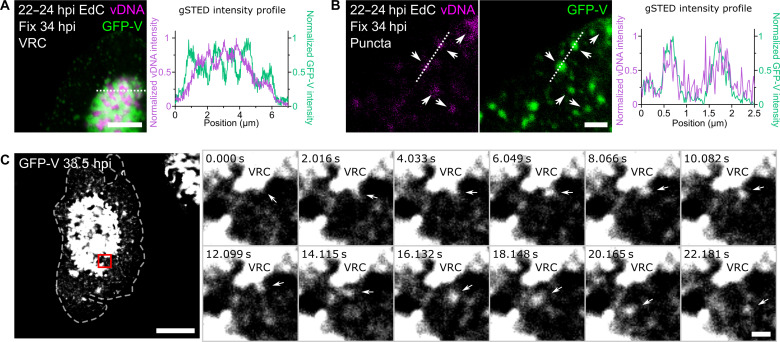
Punctuate viral assembly intermediates containing vDNA and GFP-V bubbles from the VRC periphery. (**A**) Protein V localizes to the periphery of VRC. A549 cells were infected with AdV-C2-GFP-V for 60 min, washed, pulse labeled with 2.5 μM EdC (22 to 24 hpi), washed, and fixed at 34 hpi. Samples were clicked with N_3_-AlexaFluor647 and imaged in an SP8 gSTED microscope. Data represent normalized intensities of vDNA (magenta) and GFP-V (green) across a dotted line. Scale bar, 5 μm. (**B**) gSTED analyses of nascent vDNA colocalizing with GFP-V in punctuate objects distant from the VRC. Samples were prepared and imaged as in A). Arrows indicate EdC vDNA-positive GFP-V puncta. Data represent normalized intensities of vDNA (magenta) and GFP-V (green) across a dotted line. Scale bar, 1 μm. (**C**) Nascent GFP-V puncta bubble from the VRC. A549 cells were infected with AdV-C2-GFP-V for 60 min, washed, and kept in FluoroBrite until imaging. Live-cell GFP-V signal was recorded on a single *Z* plane from 33:30 to 33:40 hpi at 0.5 Hz in an Olympus IXplore SpinSR10 spinning-disk microscope. Arrows indicate GFP-V puncta in single confocal slices. Scale bar, 10 μm. Scale bar in the zoom-in, 1 μm.

To further characterize whether the vDNA puncta represented nascent objects, we used live-cell fluorescence imaging with a replication-competent GFP-V encoding virus (AdV-C2-GFP-V), which packages the GFP-V fusion protein into virions (*32*). The bulk of GFP-V colocalized with the VRC pulsed by EdC at 22 to 24 hpi, while discrete GFP-V puncta were observed in the nucleus outside the VRC (Fig. 5A). These puncta were essentially all positive for EdC and had a size of about 100 to 400 nm, as indicated by gated stimulated emission depletion (gSTED) superresolution microscopy and line scan analyses (Fig. 5B). Live-cell imaging at 0.06 Hz demonstrated that GFP-V puncta emerged from the VRC at high frequency and quickly moved toward the nuclear periphery (fig. S6A and movies S3 and S4). Imaging at an increased acquisition frequency of 0.5 Hz revealed a nanogel-like behavior of the GFP-V puncta as they emerged from the VRC (Fig. 4C and movies S5 and S6). DFT treatment markedly reduced the amount of GFP-V puncta suggesting that the inhibition of vDNA replication impairs GFP-V puncta formation (fig. S6B and movies S7 and S8). These observations strongly suggest that the ∼400-nm-sized GFP-V puncta are nascent objects bubbling from the VRC. These objects are often located toward the nuclear periphery (Fig. 5B), whereas smaller GFP-V positive objects of about 100 nm were frequently found in the more central nucleoplasm, e.g., during very late stages of infection at 34 hpi (Fig. 5, B and C).

**Fig. 6.**
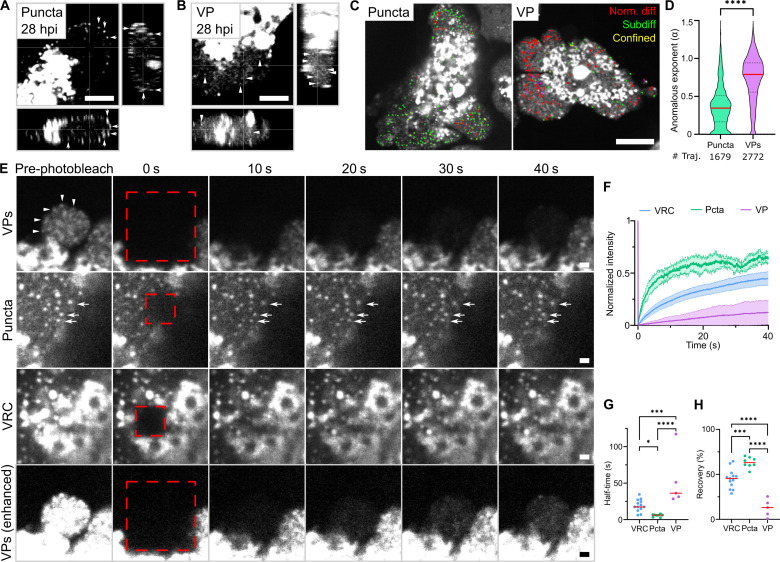
Movement, FRAP, and localization analyses of GFP-V puncta and viral particles. (**A** and **B**) GFP-V puncta localize to the nuclear periphery and viral particles are observed in the nucleoplasm at late stages of infection. Live-cell Z stacks of A549 cells infected with AdV-C2-GFP-V for 60 min, washed, and kept in FluoroBrite until imaging with an axial resolution of 0.3 μm. Arrows indicate GFP-V puncta; arrowheads indicate virus particles (VP). Images show en face projections and orthogonal slices. Scale bars, 5 μm. (**C** and **D**) Nascent GFP-V puncta near the nuclear periphery have subdiffusive movements, while viral particles have virtually unrestricted movements in the nucleus. GFP-V was recorded on single Z planes at 31.2 Hz and objects were segmented, tracked, and classified in thousand frames using TrackMate and TraJClassifier. Object trajectories from 50 consecutive frames are colored on the basis of their movement type. Data distribution is shown as violin plots. The median is shown in red. Statistical significance was determined by unpaired *t* test. ****P < 0.0001. Images are single confocal slices. Scale bar, 10 μm. (**E**) Viral particles poorly recover their GFP-V fluorescence after photobleaching, unlike nascent GFP-V puncta and VRC. A549 cells were infected with AdV-C2-GFP-V and GFP-V recorded at 31.2 Hz both before and after laser photobleaching. Arrows indicate GFP-V puncta; arrowheads indicate VP. Red dashed lines mark the photobleached region. Images are single confocal slices. Scale bars, 1 μm. (**F** to **H**) Quantitative analysis of GFP-V FRAP in VRC, nascent GFP-V puncta, and viral particles. Data were collected as described in (C). Photobleached GFP-V objects were analyzed using the jru Fiji plugins. Representative normalized FRAP curves, each starting at 1 (F), recovery half-time (tau) (G), and total fluorescence recovery (H). Pcta, puncta. Each dot in (G) and (H) is from a distinct cell (5 ≦ *n* ≦ 13). The median is shown in red. Statistical significance was determined by nonparametric ANOVA with Holm-Sidak for multiple comparisons. *P < 0.03; ***P < 0.0002; ****P < 0.0001.

To test whether the small GFP-V objects represented virus particles, we conducted single object tracking analyses by high-speed live-cell fluorescence microscopy at 31.2-Hz acquisition frequency. The GFP-V–positive small puncta homogeneously diffused through the nucleoplasm in a Brownian-like motion, as indicated by the anomalous exponent α ~ 0.8 derived from mean square displacement analyses, while the large GFP-V puncta remained subdiffusive (α ~ 0.35) and were located more toward the nuclear periphery (Fig. 6, A to D, and movies S9 and S10). The small GFP-V puncta showed a rather homogeneous distribution profile, while the larger objects had a bimodal distribution with maxima at 0 and 0.35, suggesting a static and a subdiffusive population. This was consistent with the notion that the large GFP puncta were heterogeneous, likely nascent assembly intermediates, while the small GFP-V puncta were morphologically well-defined virus particles.

**Fig. 7.**
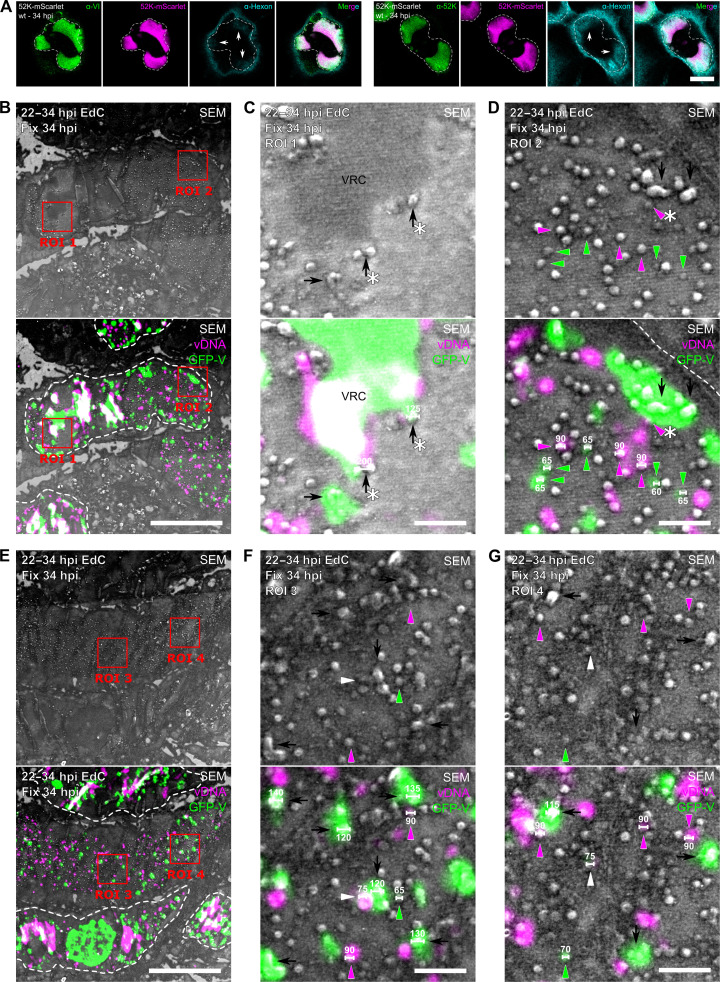
Nascent viral assembly intermediates and single virus particles were revealed by CLEM staining for vDNA and the capsid-DNA linchpin GFP-V. (**A**) AdV capsid proteins localize to the 52K compartment. A549 cells constitutively expressing a 52K-mScarlet fusion protein were infected with AdV-C5 (MOI of 3) for 60 min and fixed at 34 hpi. Samples were stained with anti-VI or anti-52K antibodies (green) and anti-hexon (cyan). Arrows indicate hexon-positive regions within the inner nuclear periphery. Images show single confocal slices. Scale bar, 10 μm. (**B** to **G**) Scanning electron microscopy (SEM) analyses and visualization of GFP-V and EdC vDNA signals by CLEM showing that nascent GFP-V puncta are virus assembly intermediates. A549 cells were infected with AdV-C2-GFP-V for 60 min, washed, labeled with 2.5 μM EdC at 22 hpi, and fixed at 34 hpi. Sectioned cell pellets were clicked with N_3_-AlexaFluor594 and imaged in a ZEISS Elyra 7 Lattice SIM^2^ microscope, followed by a Zeiss Auriga 40 CrossBeam acquisition. Images were aligned in TrakEM2 using the DAPI signal. Black arrows indicate GFP-V–positive amorphous virus assembly intermediates; black arrows with an asterisk indicate nascent GFP-V puncta bubbling from the VRC; arrowheads indicate viral particles positive for EdC (magenta) and GFP-V (green) or both markers (white); arrowheads with an asterisk indicate viral particles in close vicinity of the GFP-V–positive virus assembly intermediates. SEM object diameters are indicated in nanometers. Scale bars, 5 μm. Scale bars in the zoom-in, 500 nm.

To test whether the large GFP-V puncta comprised aggregates of virus particles, we conducted FRAP studies. About 70% of the GFP-V fluorescence in the large puncta recovered with half-time (*t*_1/2_) of about 6 s, while the recovery in the small puncta was much slower, reaching 10% with *t*_1/2_ of 30 s (Fig. 6, E to H). GFP-V in the VRC had intermediate kinetics of about 17.5 s and efficiencies of about 50%, very similar to a previous report with *L2-V-mCherry* constructs (*72*). These data show that most of the large GFV-V puncta rapidly exchange their fluorescence indicating that their GFP-V is highly accessible. Only a minor fraction of the large GFP-V objects did not recover fluorescence. In contrast, the small GFP-V objects did not exchange fluorescence indicating that their GFP-V is not exchangeable, for example, enclosed by a virion capsid. In support of the latter, two major viral capsid proteins, VI and hexon, were found to be located within the 52K condensate. Hexon was preferentially found near the nuclear rim, as shown by 52K-mScarlet fusion protein expression and indirect immunofluorescence analyses at 34 hpi (Fig. 7A). These results reinforce the notion that the majority of the large GFP-V puncta are nascent assembly intermediates, and a minority of them may contain virus particle aggregates, whereas the small GFP-V objects represent virus particles, likely formed in the 52K condensate.

### Ultrastructural analysis of vDNA- and GFP-V–positive objects reveals viral assembly intermediates

To explore the ultrastructural nature of the large and small GFP-V puncta in the nucleus, we carried out CLEM analyses. A549 cells were infected with AdV-C2-GFP-V (*32*), pulsed for nascent vDNA with EdC at 22 to 34 hpi, fixed, embedded, ultrathinly cryo-sectioned, subjected to CuAAC click reaction, stained with DAPI, and analyzed by fluorescence microscopy and scanning electron microscopy (SEM). As expected, the VRC was positive for GFP-V and EdC and devoid of SEM structures, distinguishing the compartment from the largely EdC-negative GFP-V puncta, which contained discrete SEM structures (Fig. 7B). Zoom-in views revealed irregularly shaped SEM structures on the VRC periphery positive for GFP-V and EdC (Fig. 7C). In close vicinity, distinct GFP-V–positive puncta ranging from 0.12 to ~1 μm in size harbored amorphous SEM structures of about 150 to 300 nm, suggestive of viral assembly intermediates (Fig. 7B). The SEM structures were largely negative for vDNA likely due to inaccessibility of the EdC tag for the fluorophore. In addition to VRC and GFP-V puncta, zoom-in views revealed large micrometer-sized GFP-V puncta in the periphery of the nucleus harboring irregular SEM structures (Fig. 7D, magenta arrowheads with asterisk). They were surrounded by GFP-V– and EdC-positive puncta, as well as EdC-only puncta containing regular SEM structures located toward the nuclear center (Fig. 7, E to G). These regular SEM structures were 60 to 90 nm in size and likely represented assembled virus particles that were cryo-sectioned at different Z positions, yielding variably sized profiles. Likewise, the Z position of the cryo-sections likely also gave rise to fluorescent signal heterogeneity depending on whether the vDNA core or the inner lining of the capsid with the linchpin GFP-V was cut.

**Fig. 8.**
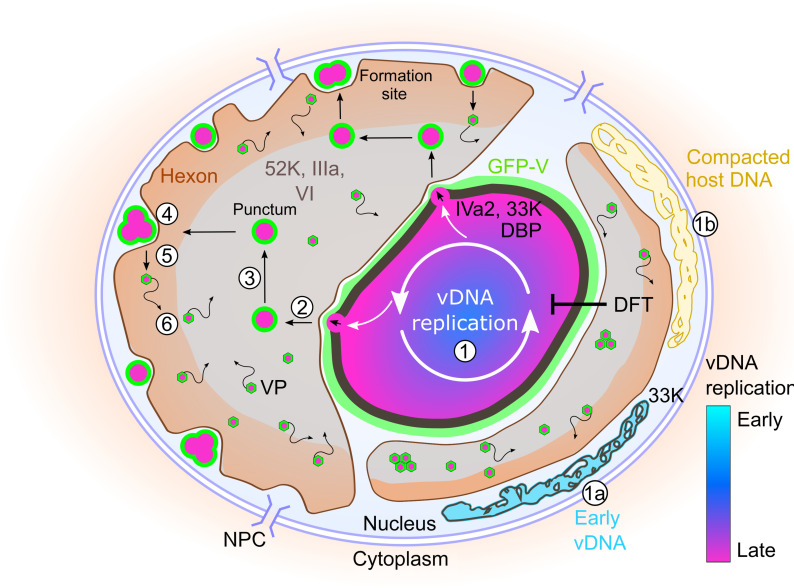
Spatiotemporal model depicting nuclear events in AdV morphogenesis, vDNA replication, nanogel-like intermediates, and formation of particles. At late stages of infection (1), early-replicated vDNA (1a) along with cellular DNA (1b) is segregated to the nuclear periphery, while late-replicated vDNA gets packaged into progeny. Depending on vDNA replication (DFT interference) and the 52K protein, nascent puncta emerge (bubble) from the VRC into the 52K/IIIa compartment (2). These nascent objects contain late-replicated vDNA, GFP-V, and incomplete virions, but not DBP. GFP-V puncta exhibit subdiffusive movements (3). In the nuclear periphery, where the capsid proteins hexon and protein VI are enriched (4), the GFP-V puncta constitute the formation site for virus particles (5), which exhibit nearly unrestricted random diffusion in the nucleoplasm (6).

Collectively, the results support a stepwise spatiotemporal model for AdV-C5 DNA packaging and particle assembly at the late stage of infection (Fig. 8). Intermediate- or late-replicated vDNA (step 1) together with the capsid-vDNA linchpin protein GFP-V emerges (bubbles) from the VRC surface. The VRC surface contains the vDNA packaging proteins IVa2 and 33K, as well as DBP engaged in vDNA replication. Bubble formation depends on vDNA replication and the 52K assembly protein arranged around the VRC together with another vDNA packaging protein, IIIa (step 2). The nanogel-like GFP-V puncta contain vDNA and virus assembly intermediates but lack DBP and are distinct from VRC. Early-replicated vDNA, which is partly positive for 33K (1a) along with cellular DNA (1b), is segregated toward the nuclear periphery, while late-replicated vDNA gets packaged into progeny virions. GFP-V puncta (nanogel-like objects) exhibit subdiffusive movements (step 3) and eventually end up in the nuclear periphery, rich in the major viral capsid protein hexon (step 4). We speculate that the GFP-V puncta in the nuclear periphery represent viral assembly intermediates from which virus particles arise (step 5). Unlike GFP-V puncta, virus particles exhibit nearly unrestricted movements in the 52K/IIIa compartment of the nucleoplasm (step 6).

## DISCUSSION

The assembly of virus particles drives disease dissemination within and between organisms and gives rise to viremia, epidemics, or pandemics. Invariably, the assembly of eukaryotic viruses requires the production of an excess of genomes and structural proteins over progeny, and the formation of de novo compartments, so-called viral factories, or inclusion bodies (*73*). A fundamental challenge has been to understand how viruses package their own genome into protein capsids excluding host DNA and RNA, notably when the genome in the VRC cannot directly interact with the host translation machinery, as in the case of DNA viruses and double-strand or negative-strand RNA viruses (*74*). The exploration of how VRCs give rise to virions has been limited by the sparsity of imaging probes for viral genomes.

Here, we developed a dual-tag genome labeling strategy for AdVs. The nature of the ethynyl and vinyl tags on the nucleoside analogs, in principle, allows adaptation to other DNA viruses. For vinyl labeling, we used a fluorogenic DNA-intercalating agent AO-6MT undergoing IEDDA reaction with VdU-tagged DNA at unprecedented sensitivity in fixed and live cells. This procedure provides distinct advantages of low invasiveness and near quantitative bioorthogonal coupling (*58*, *75*, *76*).

The VdU-AO-6MT protocol overcomes the narrow substrate specificity of endogenous nucleotide kinases in AdV E1-transformed cells thereby allowing to click-label vector DNA. VdU-AO-6MT–labeled vDNA was bright enough to allow tracking of incoming genomes in single virus particles. Unlike a recently reported pronucleotide-triphosphate analog linked to 2-trans-cyclooctene (*58*), VdU-AO-6MT is fully compatible with the serum-containing medium and not affected by carboxylesterase or lipase degradation. Viral and host DNAs labeled with VdU-AO-6MT could be observed by live-cell imaging for several hours although DNA replication eventually stalled, leading to apoptosis of infected and also uninfected cells [our results here and (*77*)].

Dual-color pulse-chase experiments combining IEDDA-VdU with CuAAC revealed that the early-replicated vDNA along with host DNA was compacted and, upon prolonged infection, became devoid of detectable active Pol II transcription and DBP-positive marks. Of note, a small fraction of early-replicated vDNA was positive for 33K which bears mRNA splicing function (*70*). This raises the possibility that a small fraction of early-replicated vDNA may serve to transcribe select viral genes late in infection. Our data also suggest that host chromatin compaction provides the volume required for the expansion of the VRC and the 52K condensate.

While negative-strand RNA viruses form compartments where both replication and assembly occur (*78*, *79*), AdV forms two kinds of membrane-free compartments, the VRC and the surrounding 52K condensate (*43*, *65*). Molecular condensates in cells can have liquid-, gel- or solid-like properties and arise by phase separation (density transitions) or gelation (percolation) (*80*). They mix and unmix nonstoichiometric monomeric and polymeric substances, including proteins, nucleic acids, and metabolites from chemical reactions (*81*–*83*). Notably, 52K is required for the formation of infectious AdV particles (*46*, *65*, *71*). The AdV VRC can be considered a dense (liquid) phase of vDNA polymers and proteins including DBP among a dilute (vapor) phase of vDNA and DBP (*43*). In there, DBP may act as a nucleoprotein stabilizing vDNA replication and phase separation. The VRC is a multicomponent object, from which single-chain vDNA molecules emerge in a process enhanced by viral packaging sequence binding proteins, such as IVa2. Of note, computational simulations with an RNA virus showed that a single high-affinity packaging site alters the collective properties of coronavirus genomic RNA by suppressing phase separation and facilitating condensation (*84*). Consistently, the AdV VRC surface is positive for DBP, IVa2, and 33K. IVa2 and 33K are involved in vDNA packaging (*67*, *85*).

Our live-cell imaging experiments of late-stage AdV infections revealed vDNA puncta emerging (bubbling) from the VRC. The puncta contained the capsid-vDNA linchpin protein V and were below 0.5 μm in size but lacked DBP implying that they are not VRC fragments. These intermediates contained 33K and the capsid linchpin protein GFP-V (*33*) and by analogy likely at least partly condensed vDNA. They were not enriched with 52K and IIIa located around the VRC. IVa2 was not detected, although it is part of assembled virions, notably at very low copy numbers (*50*). The vDNA-GFP-V intermediates remained distinct and did not fuse with the VRC implying that their components encode for precise compartmentalization.

We surmise that these assembly intermediates have nanogel-like properties, neither liquid nor solid, and emerge by processes involving low molecular weight substances, such as GFP-V, or polymers, such as vDNA. We speculate that these entities are formed through phase separation coupled to percolation (PSCP). Conceptionally, PSCP relies on components harboring sticker and spacer motives, the former engaging in specific physical crosslinks, and the latter contributing to solubility by nonspecific interactions (*80*).

By analogy with chemically fueled phase-separated droplets (*86*), the interface between VRC and 52K compartments may give rise to structures with emerging properties, such as nanogel-like viral assembly intermediates. Notably, the formation of AdV assembly intermediates depends on replication possibly implying that their segregation arises from an active process, such as vDNA elongation. This would be consistent with in vitro biophysical observations showing that chemically active droplets are positioned centrally among passive substances (*87*, *88*). Alternatively, the vDNA could be released at the cost of losing favorable interaction energy with the replication compartment. Regardless, the assembly intermediates exhibited subdiffusive movements, likely in the 52K-IIIa compartment, and possibly driven by thermal energy or by collapsing early-replicated vDNA or cellular DNA.

Of note, chromatin compaction may facilitate the accumulation of AdV assembly intermediates near the nuclear periphery, as observed at the late stages of infection. These intermediates were located in the vicinity of unassembled pools of two major virion proteins, hexon and VI, and close to vDNA and GFP-V. They contained irregular SEM structures larger than 100 nm, resembling amorphous virus particles. Unlike virions observed nearby, these assembly intermediates harbored fast exchangeable GFP-V indicating high dynamics of their contents, which may be an essential feature for virion morphogenesis. Together with their nanogel-like appearance, this makes them an attractive substance for identifying novel druggable targets.

Last, viral progeny was found to diffuse through the 52K compartment, a process which may eventually result in virion aggregate formation and well-ordered paracrystalline arrays (*89*), reminiscent of liquid crystals that eventually disperse upon rupture of the nuclear membrane during lytic virus release (*90*, *91*).

In conclusion, our study provides evidence for selective genome sequestration from the VRC into distinct assembly intermediates. We surmise that these intermediates have nanogel-like properties and emerge at the interface between an active compartment, the VRC, and a passive compartment made up of the 52K and IIIa proteins. Upon recruiting major capsid proteins in the nuclear periphery, these intermediates give rise to virions in a concurrent process, where vDNA packaging and particle assembly are coopted. The findings here open a window for exploring dynamic interactions of phase-separated compartments in viral morphogenesis and beyond.

## MATERIALS AND METHODS

### Cell culture and virus production

HeLa-ATCC, A549, HEK293T, and HER911 cells were maintained in Dulbecco’s modified Eagle’s medium (DMEM; Gibco) supplemented with nonessential amino acids (Thermo Fisher Scientific) and 10% fetal calf serum (FCS; Gibco). Polyclonal A549 cells constitutively expressing 52K-mScarlet were produced through lentiviral transduction of a modified pLVX-Puro construct, containing the 52K-mScarlet fusion protein under the expression of a CMV promoter, and selected with puromycin (2 μg/ml) for 10 days. During infection experiments, the medium was additionally supplemented with penicillin (100 U/ml) and streptomycin (100 μg/ml). The cells were grown at 37°C in a 5% CO_2_ atmosphere for no longer than 20 passages.

All AdVs were grown in A549 cells and purified over two cesium chloride gradients as previously described (*92*). AdV-C5 (wt300) has been previously described (*93*). AdV-C5_Δ52K was provided by M. Imperiale (University of Michigan Medical School, USA) (*71*). AdV-C2-GFP-V was used as described (*32*). VdU-labeled AdV-C5 was produced as previously described (*21*) with the following modifications: VdU was added to the cells at 4 hpi to a final concentration of 50 μM and kept until harvest and purification.

### Transmission electron microscopy

For the analysis of purified AdV particles, 5 μl of glycerol-free purified virus was mounted onto carbon-coated grids for 5 min. Grids were washed three times with distilled water and stained for 30 s with 10 μl of a 2% aqueous uranyl acetate solution. Samples were imaged in a CM100 transmission electron microscope at 80 keV (Thermo Fisher Scientific, Eindhoven, Netherlands).

### Antibodies

The following primary antibodies against AdV were used: rabbit anti-VI, immunofluorescence (IF) 1:2000 (*94*); mouse anti–AdV-C5 hexon 9C12, IF 1:40 (*95*); mouse anti-DBP, IF 1:150 [provided by A. Levine (*96*)]; rabbit anti-IVa2, IF 1:100 [provided by P. Hearing (*85*)]; rabbit anti-33K, IF 1:250 [provided by P. Hearing (*67*)]; rabbit anti-IIIa, IF 1:1000 [provided by P. Hearing (*97*)]; and rabbit anti-52K, IF 1:1000 [provided by P. Hearing (*85*)]. Mouse anti–phospho-Ser5-RNA Pol-II, IF 1:1000 (ab5408, Abcam).

### Virus titration

Ten thousand cells were seeded per well in a black 96-well imaging plate. On the following day, viruses were diluted in the infection medium (DMEM supplemented with 2% FCS, nonessential amino acids, and penicillin/streptomycin). The culture supernatant was aspirated, and cells were inoculated with 100 μl of virus dilution for 60 min at 37°C; subsequently, the inoculum was removed, and cells were supplemented with 100 μl of a fresh infection medium. The cells were fixed at 24 hpi with 3% paraformaldehyde (PFA) in phosphate-buffered saline (PBS) for 15 min at room temperature (RT). The remaining PFA was quenched with 25 mM NH_4_Cl diluted in PBS for 10 min, followed by permeabilization with 0.5% Triton X-100 in PBS for 5 min. Cells were stained with rabbit anti–protein VI (*94*) diluted in a blocking buffer (10% goat serum in PBS) for 1 hour at 4°C. After three washes for 3 min each in PBS, cells were stained with a secondary antibody (goat anti-rabbit AlexaFluor488, Thermo Fisher Scientific) diluted in a blocking buffer containing DAPI (1 μg/ml) at RT for 1 hour. After three more washes for 4 min in PBS, cells were imaged in a Molecular Devices high-throughput microscope (IXMc) in wide-field mode with a 20× objective. For quantification of infection with CellProfiler (*98*), nuclei were segmented according to the DAPI signal, and the intensity of protein VI over the nuclear mask was measured. MOI was defined by the probability of infection based on AdV protein VI expression according to the adapted Poisson distribution by Ellis and Delbrück (*99*).

### Virion analysis on glass coverslips

Glass coverslips were coated with a 120-μl drop of poly-l-lysine (0.1 mg/ml; Sigma-Aldrich) for 30 min at RT. In the meantime, 0.3 μg of purified virus was diluted in Hepes buffer (25 mM Hepes, 150 mM NaCl, and 1 mM MgCl_2_ in ddH_2_O) and incubated for 12 min at on ice or at 45°C in a water bath. Excess poly-l-lysine was removed, and mock or heat-treated viruses were added on the poly-l-lysine–coated coverslips for 30 min at RT and fixed with 3% PFA at RT for 15 min. Samples were quenched with NH_4_Cl for 10 min, stained with mouse anti-hexon 9C12 (*95*) in a blocking buffer at 4°C for 1 hour, and stained with secondary antibody goat anti-mouse/rabbit AlexaFluor680 (Thermo Fisher Scientific) in a blocking buffer at RT for 1 hour. Samples were clicked with either IEDDA or CuAAC reactions as described above. Coverslips were mounted onto slides in ProLong Gold Antifade Mountant (Thermo Fisher Scientific), let dry over two nights, and imaged in a Leica SP8 FALCON CLSM as described.

### Virus progeny production and TCID_50_ assay

Fifteen thousand cells were seeded per well in a transparent 96-well plate. On the following day, viruses were diluted in the infection medium to a final MOI of 1.5. The cell culture supernatant was aspirated, and cells were inoculated with 100 μl of virus dilution at 37°C for 120 min; subsequently, the inoculum was removed, and cells were supplemented with DMSO or vinyl-modified nucleosides diluted in a fresh infection medium and kept until the cytopathic effect was observed (~72 hpi for AdV-C5). Supernatant and cells were collected, freeze thawed twice in liquid nitrogen, snap frozen a third time, and kept at −80°C until use; upon use, samples were thawed a third time and debris was removed by centrifugation at 4000*g* for 3 min.

For progeny titration, 10,000 cells were seeded per well in a transparent 96-well plate. On the following day, the collected virus was diluted in an infection medium, added on top of the cells, and incubated at 37°C. Seven days pi cells were fixed by adding 16% PFA on top to reach a final concentration of 3% at RT for 30 min. Fixative was discarded and cells were stained with 40 μl of crystal violet staining solution [crystal violet (2.5 mg/ml) and 10% MeOH in double-distilled H_2_O] at RT for 60 min. Excess staining was washed away multiple times in ddH_2_O, wells were scored as infected when the cell monolayer was not intact, and median tissue culture infectious dose (TCID_50_) was determined according to the Spearman-Kärber method.

### IEDDA click chemistry and vDNA analysis

Eighty thousand A549 cells were seeded on 12-mm glass coverslips in a 24-well plate. On the following day, viruses were diluted in the infection medium to a final MOI of 3. The cell culture supernatant was aspirated, and cells were inoculated with 300 μl of virus dilution for 60 min at 37°C; subsequently, the inoculum was removed, and fresh infection media were added to the cells. Infected cells were tagged with 50 μM VdU for 4 hours in a 2% FCS-containing medium before fixation. Noninfected samples were labeled with 50 μM VdU for the entire duration of the experiment. Cells were fixed with 3% PFA for 15 min at RT, quenched with 25 mM NH_4_Cl for 10 min, and permeabilized with 0.5% Triton X-100 for 5 min. Cells were stained with mouse anti-DBP (provided by A. Levine) (*96*) in a blocking buffer for 1 hour at 4°C. Cells were washed three times for 3 min in PBS and stained with secondary antibody goat anti-mouse AlexaFluor594 (Thermo Fisher Scientific), unless specified, in a blocking buffer at RT for 1 hour. After primary and secondary antibody incubation, the coverslips were inverted onto a 35-μl droplet of freshly prepared IEDDA click reaction mix (50 μM AO-6MT in PBS) at 37°C for 4 hours. Samples were stained with DAPI and imaged in a Leica SP8 FALCON CLSM. Three-dimensional image stacks were analyzed using Imaris10 (Oxford Instruments, Oxon, UK). VdU-AO-6MT objects were segmented within the DAPI-delimited volume and classified into VRC and puncta based on a voxel size threshold of 50 (image voxel size: *X*, 0.18 μm; *Y*, 0.18 μm; *Z*, 0.5 μm). Images were batch processed and segmentation was manually validated. Overlap and distances were computed between the volumes within the nucleus.

### CuAAC click chemistry and vDNA analysis

Eighty thousand A549 cells were seeded on 12-mm glass coverslips in a 24-well plate. On the following day, cells were infected as previously described. Infected cells were labeled with 2.5 μM EdC 4 hours before fixation unless specified. Noninfected samples were labeled with 2.5 μM EdC for the entire duration of the experiment. Cells were fixed, quenched, and permeabilized as previously described. Cells were stained with mouse anti-DBP (B6–8; provided by A. Levine) in a blocking buffer for 1 hour at 4°C and stained with secondary antibody goat anti-mouse AlexaFluor594 (Thermo Fisher Scientific), unless specified, in a blocking buffer for 1 hour at RT. After primary and secondary antibody incubation, the coverslips were inverted onto a 30-μl droplet of freshly prepared CuAAC click reaction mix [10 μM N_3_-AlexaFluor647 (Thermo Fisher Scientific), 1 mM CuSO_4_, 10 mM aminoguanidine (Sigma-Aldrich), 1 mM tris(3-hydroxypropyltriazolylmethyl)amine (Sigma-Aldrich), and 10 mM sodium ascorbate in PBS] at RT for 2 hours. Samples were stained with DAPI and imaged in a Leica SP8 FALCON CLSM. Three-dimensional image stacks were analyzed using Imaris10 (Oxford Instruments, Oxon, UK). EdC-N_3_-AlexaFluor objects were segmented within the DAPI-delimited volume and classified into VRC and puncta based on a voxel size threshold of 50 (image voxel size: *X*, 0.18 μm; *Y*, 0.18 μm; *Z*, 0.5 μm). Images were batch processed and segmentation was manually validated. Overlap and distances were computed between the volumes within the nucleus.

### Dual-label click chemistry

Eighty thousand A549 cells were seeded on coverslips. On the following day, viruses were diluted in the infection medium to a final MOI of 3. The cell culture supernatant was aspirated, and cells were inoculated with 300 μl of virus dilution at 37°C for 60 min; subsequently, the inoculum was removed, and fresh infection media were added to the cells. Infected cells were pulse labeled with 2.5 μM EdC for 4 hours at specified time points, EdC was washed out two times with PBS, and a fresh infection medium was added. Cells were labeled with 50 μM VdU for 4 hours before fixation unless specified. Nucleoside analogs added to the cells in the reverse order were used at the same concentrations. Cells were fixed, quenched, and permeabilized as previously described. Samples were stained with mouse anti-DBP and secondary goat anti-mouse AlexaFluor594 as previously described unless specified. Samples were firstly IEDDA-clicked flowed-up by the CuAAC click reaction as previously described. Samples were stained with DAPI, mounted onto slides in ProLong Gold Antifade Mountant, and imaged in a Leica SP8 FALCON CLSM. Three-dimensional image stacks were analyzed using Imaris10 as previously described.

### Virus entry and vDNA uncoating

Eighty thousand HeLa cells were seeded on 12-mm glass coverslips in a 24-well plate. On the following day, cells were incubated with ~100 bound particles of the virus in the infection medium at 37°C for 30 min as described (*33*). Inocula were removed, and cells were fixed or incubated in a fresh infection medium at 37°C for 150 min. Cells were fixed, quenched, and permeabilized as previously described. Cells were stained with mouse anti-hexon 9C12 in a blocking buffer at 4°C for 1 hour, stained with secondary antibody goat anti-mouse/rabbit AlexaFluor680 (Thermo Fisher Scientific) in a blocking buffer at RT for 1 hour, and IEDDA-clicked as described above. Coverslips were mounted onto slides in ProLong Gold Antifade Mountant, let dry over two nights, and imaged in a Leica SP8 FALCON CLSM as described.

### Confocal microscopy

A Leica SP8 FALCON or SP8 gSTED CLSM was used in all experiments, in which single viral genomes and subnuclear objects were imaged. Imaging was performed with a ×63 magnification oil objective with a numerical aperture (NA) of 1.40 and a zoom factor of 2, with a pixel size of 0.181 μm. Z stacks were captured with a step size of 0.3 to 0.5 μm to capture the entire cell, and the size of the pinhole was 1 Airy unit. For the acquisition of virus particles on coverslips, the pinhole was 6.28 Airy units. Leica hybrid detectors were used for each channel.

### Confocal spinning-disk live microscopy, particle tracking, and FRAP

Ten thousand A549 cells were seeded in a black glass-bottom 96-well imaging plate. On the following day, cells were inoculated with the virus at an MOI of 3 for 60 min at 37°C; subsequently, the inoculum was removed, and cells were supplemented with 100 μl of a fresh infection medium. Noninfected cells were labeled with VdU upon seeding and washed before infection. Infected cells were pulse labeled with VdU at 16 to 20 hpi. Subsequently, the medium was removed, and samples were clicked with AO-6MT in FluoroBrite (Thermo Fisher Scientific) containing Hoechst 33342 as indicated. Imaging started 1 hour after AO-6MT addition at a frequency of two to four frames per hour in a microscope IXMc spinning-disk microscope in confocal mode with a 20× objective and a 60-μm pinhole. Samples were fixed, quenched, permeabilized, stained, and re-imaged as described above. Samples were quantified with CellProfiler as described above.

For the imaging of viral cores, 15,000 A549 cells were seeded in eight-well μ-slides (Ibidi) over two nights. Cells were inoculated with AdV-C2-GFP-V at a final MOI of 3 in 180 μl of infection media at 37°C for 60 min, the inoculum was removed, and cells were supplemented with 300 μl of a fresh infection medium. Media were replaced with mock or 2.5 μM DFT-supplemented FluoroBrite (Thermo Fisher Scientific) at the specified times. Imaging started at the specified times and frame frequencies in an Olympus IXplore SpinSR10 spinning-disk microscope with a 100× objective in silicon oil. GFP-V objects were tracked on the high-frequency acquired images (32 ms per frame) using Fiji’s plugin TrackMate. Particle trajectories were then classified using TraJClassifier.

FRAP of the GFP-V objects was performed in an Olympus IXplore SpinSR10 spinning-disk microscope with a Rapp OptoElectronics photomanipulation unit using a 405-nm laser at 10% of its maximum intensity. Fluorescence recovery was recorded for at least 60 s. Fluorescence recovery was analyzed using the following Fiji plugins: create spectrum jru, combine all trajectories jru, normalize trajectories jru, batch frap fir jru, and average trajectories jru.

### Superresolution gSTED microscopy

Eighty thousand A549 cells were seeded on coverslips. On the following day, AdV-C2-GFP-V was diluted in the infection medium to a final MOI of 3. Cells were infected and pulse labeled with EdC as described previously. Cells were fixed with 2% PFA for 15 min at RT, quenched with 25 mM NH_4_Cl for 10 min, and permeabilized with 0.1% Triton X-100 for 10 min. Cells were blocked with a blocking buffer at 4°C for 1 hour. Samples were CuAAC-clicked with an N_3_-AlexaFluor647 as described previously. Coverslips were mounted onto slides in ProLong Gold Antifade mounting medium, let dry over two nights, and imaged in a Leica SP8 inverse STED 3X CLSM. Imaging was performed with a 100× magnification oil objective with NA of 1.4 and a zoom factor of 6, with a pixel size of 19 nm and gating from 1.5 to 6 ns. A 592-nm depletion laser was used for the GFP, and a 775-nm depletion laser was used for N_3_-AlexaFluor647.

### Correlative light and electron microscopy

Four hundred thousand A549 cells per well were seeded in a six-well plate. On the following day, AdV-C2-GFP-V was diluted in the infection medium to an MOI of 3. Cells were infected at 37°C for 60 min, washed, incubated at 37°C, and supplemented with 2.5 μM EdC at 22 hpi until fixation in a mixture of 0.1 M sodium cacodylate buffer, 4% formaldehyde (Electron Microscopy Sciences, #15710), and 0.1% glutaraldehyde (Electron Microscopy Sciences, #16220) overnight at 4°C. Cells were collected by scraping in PBS/0.5% bovine serum albumin using a rubber policeman, washed, centrifuged, embedded in warm 12% gelatin at 40°C for 10 min, and solidified at 4°C for 30 min. Embedded cell pellets were cryoprotected in 2.3 M sucrose and stored at 4°C. CLEM was carried out as described (*100*). Briefly, CLEM samples were frozen in liquid nitrogen and sectioned with a cryo-ultramicrotome (Ultracut EM Artos 3D, Leica Microsystems) using a cryo immuno diamond knife (Diatome). Ultrathin sections (110 nm) were transferred to a 7 mm–by–7 mm silicon wafer (Si-Mat Silicon Materials, Kaufering, Germany) and stored at 4°C. The ultrathin sections were incubated with PBS at 0°C for 20 min, followed by two washing steps with PBS each at RT for 2 min to dissolve the cryoprotectant. The wafers were incubated with CuAAC click reaction reagents (see above) at RT in the dark for 90 min, washed in PBS three times for 2 min, followed by a 1-min incubation with DAPI as described (*38*). Before imaging, the wafers were incubated with a 1:1 mixture of 87% glycerin (Sigma-Aldrich) and PBS twice for 10 s and transferred to a glass-bottom petri dish (Ibidi, Gräfelfing, Germany) sections facing down and imaged with a ZEISS Elyra 7 Lattice SIM^2^ microscope equipped with a 63× NA 1.4 Plan-Apochromat objective lens. After light microscopy imaging, the wafers were incubated with 2% methylcellulose, centrifuged, and imaged with a scanning electron microscope (Auriga 40, CrossBeam; Carl Zeiss Microscopy, Jena, Germany) at low acceleration voltage (0.8 keV) using simultaneously InLens and ESB detectors at pixel size of 3 nm and with a dwell time of 100 μs. Lattice SIM^2^ was performed on the fluorescence images using the “Zen for Elyra” software (Zeiss). Fluorescence images were aligned with multispectral fluorescence beads using the “Zen for Elyra” lateral alignment mode. Image correlation was performed manually with the plugin TrakEM2 using the DAPI signal as a reference (*101*).

### Statistical analyses

All graphs were generated using RStudio or GraphPad Prism and display means ± SD unless stated otherwise. Statistical tests used are indicated in the figure legends (ns, not significant; *P < 0.05, **P < 0.01, and ***P < 0.001).
