# Supplementary material for: Stepwise virus assembly in the cell nucleus revealed by spatiotemporal click chemistry of DNA replication
Source: Sci Adv. 2024 Oct 25;10(43):eadq7483. doi: 10.1126/sciadv.adq7483 (PMC11506174; doi:10.1126/sciadv.adq7483)
Supplement: Supplementary file 1 — Figs. S1 to S6 Legends for movies S1 to S10 [file sciadv.adq7483_sm.pdf]

Science Advances
AAAS

Supplementary Materials for

Stepwise virus assembly in the cell nucleus revealed by spatiotemporal click chemistry of DNA replication

Alfonso Gomez-Gonzalez *et al.*

Corresponding author: Urs F. Greber, urs.greber@mls.uzh.ch

Sci. Adv. **10**, eadq7483 (2024)
DOI: 10.1126/sciadv.adq7483

The PDF file includes:

Figs. S1 to S6
Legends for movies S1 to S10

Other Supplementary Material for this manuscript includes the following:

Movies S1 to S10

Supplementary Figures

Figure S1

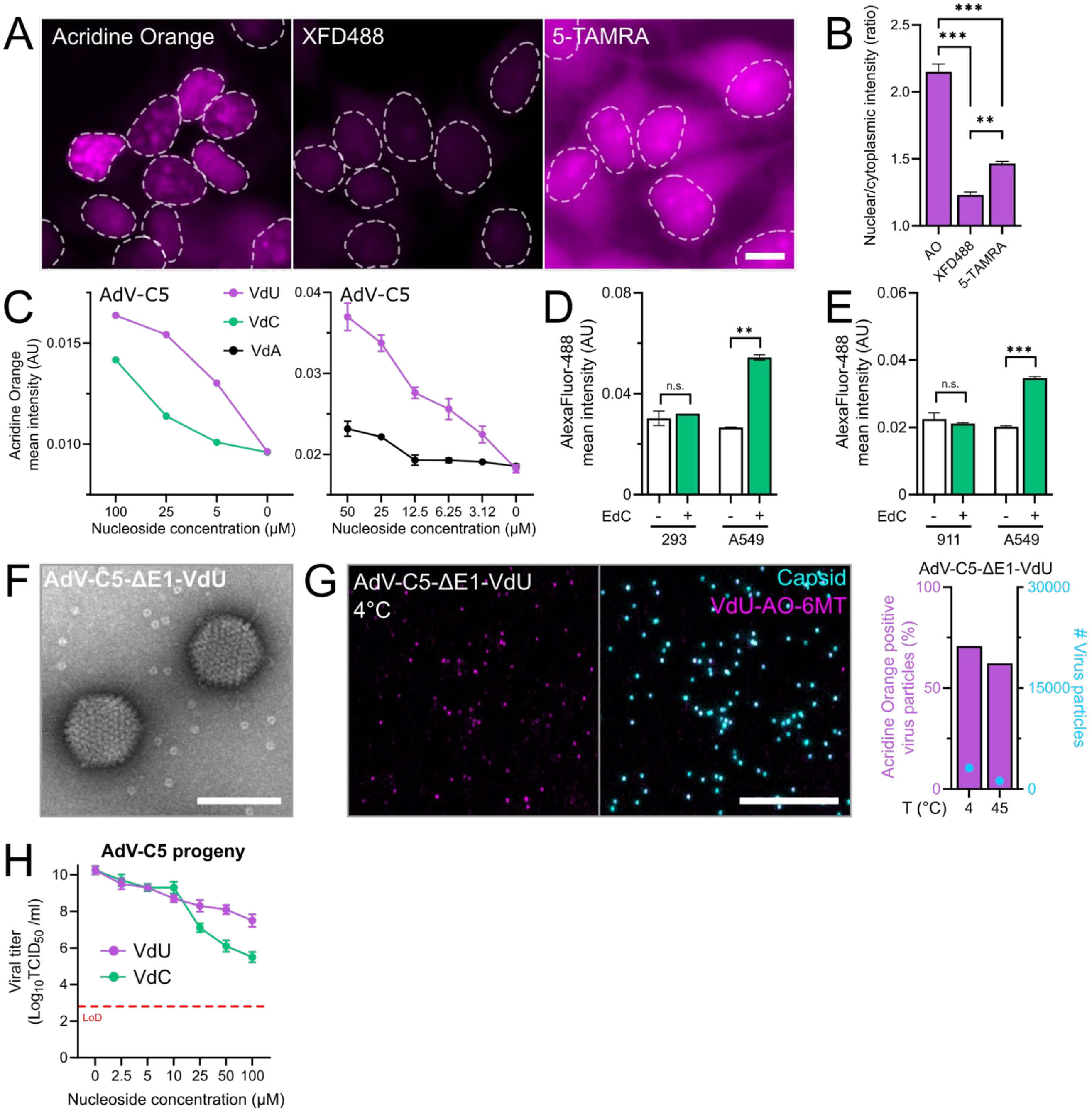

Figure S1: The vinyl-modified nucleosides VdU and VdC are incorporated into AdV-C5 wild-type or AdV-C5 vector DNA with moderate effects on viral progeny formation (related to Fig. 1)

(A) Comparative analysis of tetrazine coupling to VdU-labeled DNA. A549 cells were labeled with VdU for 24 h, fixed and clicked with either XFD488-6-MT or 5-TAMRA-6-MT. Dashed lines indicate the nuclear rim. Scale bar, 10 µm.

(B) Quantitative analysis of the nuclear to cytoplasmic signal of different fluorophore-coupled tetrazines. Samples were prepared as described in A). Nuclei stained with DAPI. Data represent means ± SD. Statistical significance was determined by non-parametric ANOVA with Holm-Sidak for multiple comparisons. **, $p<0.002$; ***, $p<0.0002$.

(C) Quantitative analyses of vinyl-modified nucleoside incorporation in A549 cells infected with AdV-C5 (MOI 1.5) and clicked with AO-6MT. Nuclei were segmented based on DAPI signal and the AO signal was quantitated in the nuclear area. Data represent means ± SD.

(D, E) Quantitative analyses of EdC incorporation into HEK-293 and HER-911 cells. Samples were prepared as described in Fig. 1D and E. Data represent means ± SD of the N_3-AlexaFluor488 signal over the DAPI-stained nuclei. Statistical significance was determined by non-parametric ANOVA with Holm-Sidak for multiple comparisons. **, $p<0.0021$; ***, $p<0.0002$; ns, non-significant.

(F) Electron micrograph of negatively stained VdU-labeled AdV-C5_ΔE1 vector particles. Scale bar, 100 nm.

(G) Representative images and quantitative analysis of purified AdV-C5_ΔE1-VdU particles stained with AO-6MT. Virions were bound to poly-lysine-coated coverslips, fixed, stained with the anti-hexon 9C12 antibody and clicked with AO-6MT. Data represent percentage of AO-6MT-positive virus particles. Scale bar, 10 µm.

(H) AdV-C5 progeny production in presence of vinyl-modified nucleosides. A549 cells were inoculated with AdV-C5 at 37°C for 2 h, washed in medium and labeled with vinyl-modified nucleosides at 8 hpi. Progeny virus was collected at 72 hpi and titrated by $TCID_{50}$. Data represent Spearman-Kärber $TCID_{50}$ values ± SD.

Figure S2

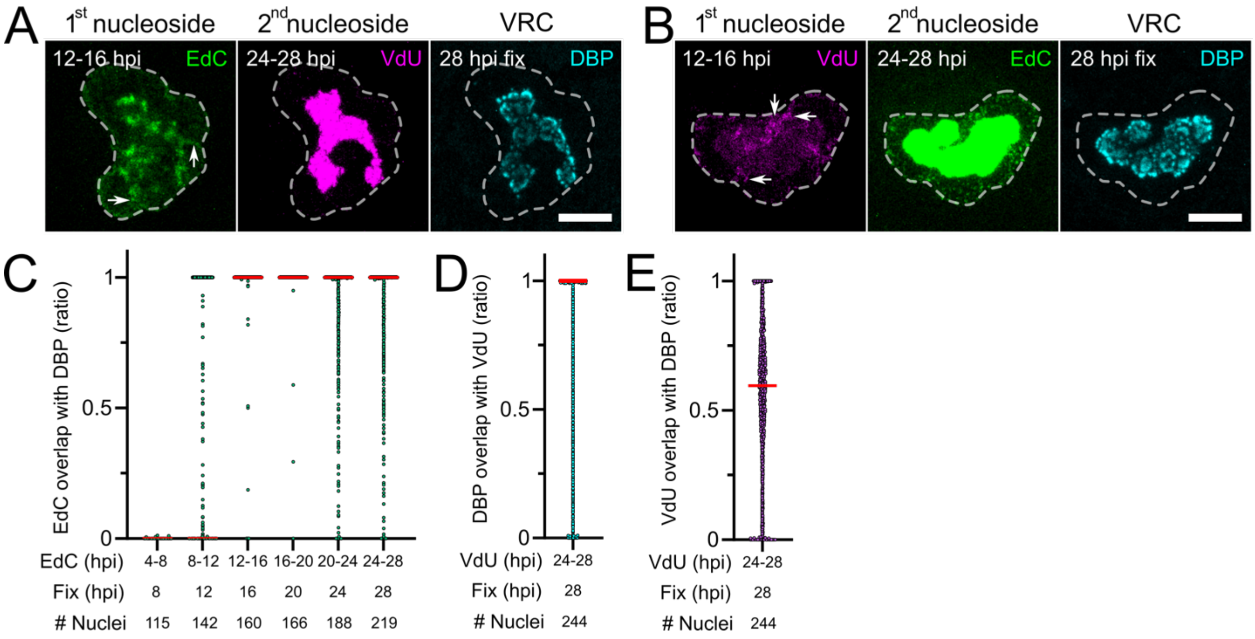

Figure S2: VdU-AO-6MT and EdC-N3-AlexaFluor identify distinct pools of vDNA in infection (related to Fig. 2)

(A, B) Comparative analysis of EdC and VdU incorporation in AdV-C5 infected cells. A549 cells were infected with AdV-C5 (MOI 3) for 60 min, washed, pulsed with 2.5 µM EdC at 12-16 hpi, pulsed with 50 µM VdU at 24-28 hpi (A) or vice versa (B) and fixed at 28 hpi. Arrows indicate early replicated vDNA outside of the VRC. Samples were stained with anti-DBP antibodies (cyan), clicked with AO-6MT (magenta) as well as N_3-AlexaFluor647 (green). Scale bar, 10 µM.

(C) Quantification of EdC-labeled vDNA overlap with DBP. Samples were prepared as described in Fig. 2B. EdC-labeled vDNA objects were 3D-segmented based on the EdC-Alexa647 signal. Overlap was computed with respect to 3D-segmented DBP objects. Each data point represents an EdC-labeled vDNA object. Median indicated in red.

(D) Quantitative analysis of DBP overlap with VdU-labeled vDNA. Samples were prepared as described in Fig. 2B. DBP objects were 3D-segmented based on the DBP signal. Overlap was computed with respect to 3D-segmented VdU-labeled vDNA objects. Each data point represents a DBP object. Median indicated in red.

(E) Quantitative analysis of VdU-labeled vDNA overlap with DBP. Samples were prepared as described in Fig. 2B. VdU objects were 3D-segmented based on the VdU-AO-6MT signal. Overlap was computed with respect to 3D-segmented DBP objects. Each data point represents a VdU-labeled vDNA object. Median indicated in red.

Figure S3

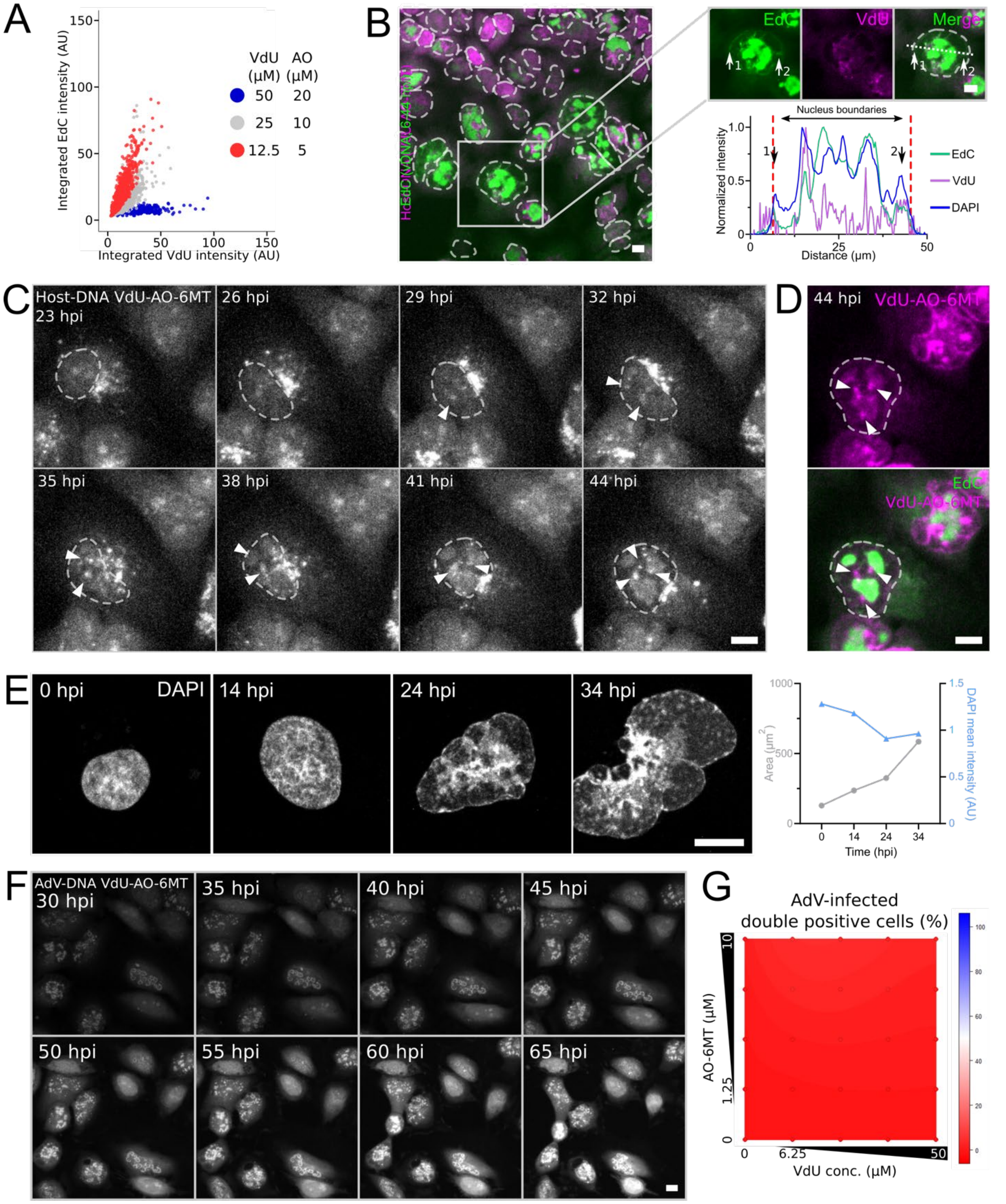

Figure S3: VdU-AO-6MT labels viral and host DNA and stalls viral replication (related to Fig. 2)

(A) Dose-dependent incorporation of EdC during AdV-C5 infection in VdU-AO-6MT DNA labeled cells. A549 cells were grown in different concentrations of VdU, followed by

infection with AdV-C5 (MOI 3) for 60 min, washed and labeled with AO-6MT (concentrations adjusted to the VdU concentrations) and 2.5 µM EdC in FluoroBrite at 6 to 44 hpi. Cells were fixed 44 hpi, clicked with N_3-AlexaFluor647 and stained with DAPI. Data represent the integrated VdU-AO-6MT and EdC-AlexaFluor647 intensities measured over the DAPI-stained nuclei. Each data point represents one nucleus.

(B) Comparative analysis of the cellular and viral DNA distribution in AdV-C5 infection. Samples were prepared as described in the previous panel with 25 µM VdU and 10 µM AO-6MT. Dashed lines indicate nuclear periphery. Zoomed nuclei show EdC signal localizing at the nuclei periphery. Data represent EdC-labeled vDNA, VdU-labeled cell DNA and DAPI normalized intensities measured over the white dotted line. Arrows indicate chromatin condensation events of viral origin. Images are maximum projections. Scale bar, 10 µm.

(C, D) Live cell imaging of VdU-AO-6MT labeled host DNA during AdV-C5 infection, including the 44 hpi endpoint. Samples were prepared as described in panel A) with 25 µM VdU and 10 µM AO-6MT. Live imaging was from 8-44 hpi at a frequency of four frames per hour. Arrowheads indicate cellular chromatin condensation events. The prominent extranuclear signals represent acidic compartments, where AO-6MT tends to accumulate over time. Images are maximum projections. Scale bar, 10 µm.

(E) Changes in nuclear morphology in the course of AdV-C5 infection. A549 cells were infected with AdV-C5 (MOI 3) for 60 min, washed, fixed at the indicated times pi and stained with DAPI. Scale bar, 10 µm. Data represent the nuclear area and the DAPI mean intensity of each shown nuclei.

(F) Live cell imaging of AdV-C5 VRCs. A549 cells were inoculated with AdV-C5 (MOI 3) for 60 min, washed and incubated with 25 µM VdU from 18-22 hpi, followed by AO-6MT (10 µM) addition in FluoroBrite containing Hoechst DNA dye. Live imaging was from 23-65 hpi at a frequency of two frames per hour. Images shown are maximum projections. Scale bar, 50 µm.

(G) Quantitative analysis of EdC incorporation in VdU-AO-6MT labeled AdV-C5 infection. A549 cells were infected with AdV-C5 (MOI 3) for 120 min, washed, pulse-labeled with 25 µM VdU at 16-20 hpi, clicked with 10 µM AO-6MT at 20-24 hpi and labeled with 2.5 µM EdC at 24-28 hpi. Cells were fixed at 28 hpi, clicked with N_3-AlexaFluor647 and stained with DAPI. Data represent percentage of double nucleoside-positive DAPI-stained nuclei across various VdU and AO-6MT concentrations.

Figure S4

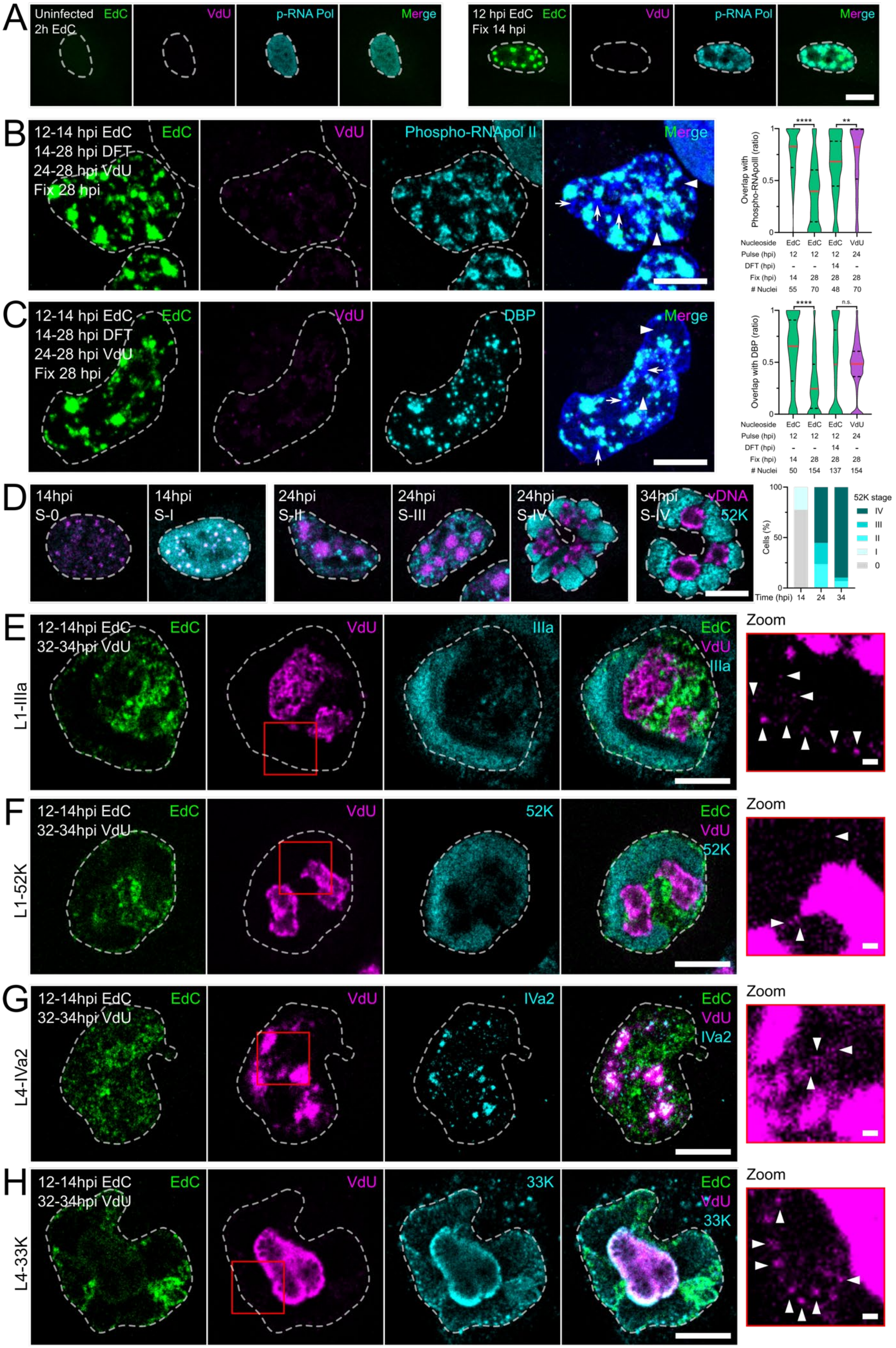

A
Uninfected 2h EdC
EdC
VdU
p-RNA Pol
Merge
12 hpi EdC Fix 14 hpi
B
12-14 hpi EdC
14-28 hpi DFT
24-28 hpi VdU
Fix 28 hpi
Phospho-RNApol II
Overlap with Phospho-RNApolII (ratio)
Nucleoside
Pulse (hpi)
DFT (hpi)
Fix (hpi)
Nuclei
C
DBP
Overlap with DBP (ratio)
n.s.
D
14hpi S-0
14hpi S-I
24hpi S-II
24hpi S-III
24hpi S-IV
34hpi S-IV
vDNA
52K
52K stage
Cells (%)
Time (hpi)
E
12-14hpi EdC
32-34hpi VdU
IIIa
L1-IIIa
Zoom
F
52K
L1-52K
G
IVa2
L4-IVa2
H
33K
L4-33K

Figure S4: Loss of active RNA-Pol-II and DBP from VRC requires ongoing vDNA replication, and recruitment of the DNA packaging proteins IVa2 and 33K to the VRC but not 52K and IIIa, which localize around the VRC (related to Fig. 3)

(A) Distribution of p-RNA-Pol-II across AdV-C5 infected A549 cells. Samples were prepared as described in Fig. 3A. Images are maximum projections. Scale bar, 10 µm. For additional VdU-AO-6-MT control and background staining, see F1G, H and SF1A, B, as well as the raw images at https://doi.org/10.5281/zenodo.12832548.

(B) Representative images and quantitative analysis of active RNA-Pol-II and DBP on VRC depending on vDNA replication. Samples were infected and labeled as described in Fig. 3A. DNA replication was inhibited with 2.5 µM 3′-deoxy-3′-fuorothymidine (DFT) at 14-28 hpi. Samples were stained with p-RNA Pol-II (cyan) and DAPI (blue), and clicked with AO-6MT (magenta) and N_3-AlexaFluor594 (green). Data distribution is shown as violin plots. Median indicated in red. Statistical significance was determined by non-parametric ANOVA with Holm-Sidak for multiple comparisons. **, p<0.0021; ****, p<0.0001. Arrows indicate dense chromatin regions stained with DAPI; arrowheads indicate chromatin-free nuclear regions. Images are maximum projections. Scale bar, 10 µm.

(C) Representative images and quantitative analysis VRC fragmentation in absence of ongoing vDNA replication. Samples were prepared as described in panel B). Samples were stained with anti-DBP (cyan) and DAPI (blue), and clicked with AO-6MT (magenta) and N_3-AlexaFluor594 (green). Statistical analysis was performed as indicated in panel B). p<0.0002; ****, p<0.0001; ns, non-significant. Arrows indicate chromatin-dense nuclear regions; arrowheads indicate chromatin-free nuclear regions. Images are maximum projections. Scale bar, 10 µm.

(D) Subnuclear distribution of 52K in the course of AdV infection. Cells were infected with AdV-C5 at MOI 3. Specimens were labeled with 2.5 µM EdC at 12-14 hpi or 22-24 hpi and with 50 µM VdU at 32-34 hpi, stained with anti-52K (cyan), and clicked with either AO-6MT or N_3-AlexaFluor594. Nuclear 52K stages were classified manually as described in Ref 65.

(E-H) Localization of viral assembly factors IIIa (E), 52K (F), IVa2 (G) and 33K (H) with respect to VRC and early- or late-replicated vDNA at late stages of infection (34 hpi). Specimens were labeled with 2.5 µM EdC at 12-14 hpi and with 50 µM VdU at 32-34 hpi, stained with diverse antibodies (cyan), and clicked with AO-6MT (magenta) and N_3-AlexaFluor594 (green). Red squares show cropped-in regions at higher contrast to highlight vDNA puncta. Arrowheads indicate single VdU-labeled puncta (labeled at 32 hpi). Images are single confocal slices. Scale bar, 10 µm. Scale bar in zoom-in, 1 µm.

Figure S5

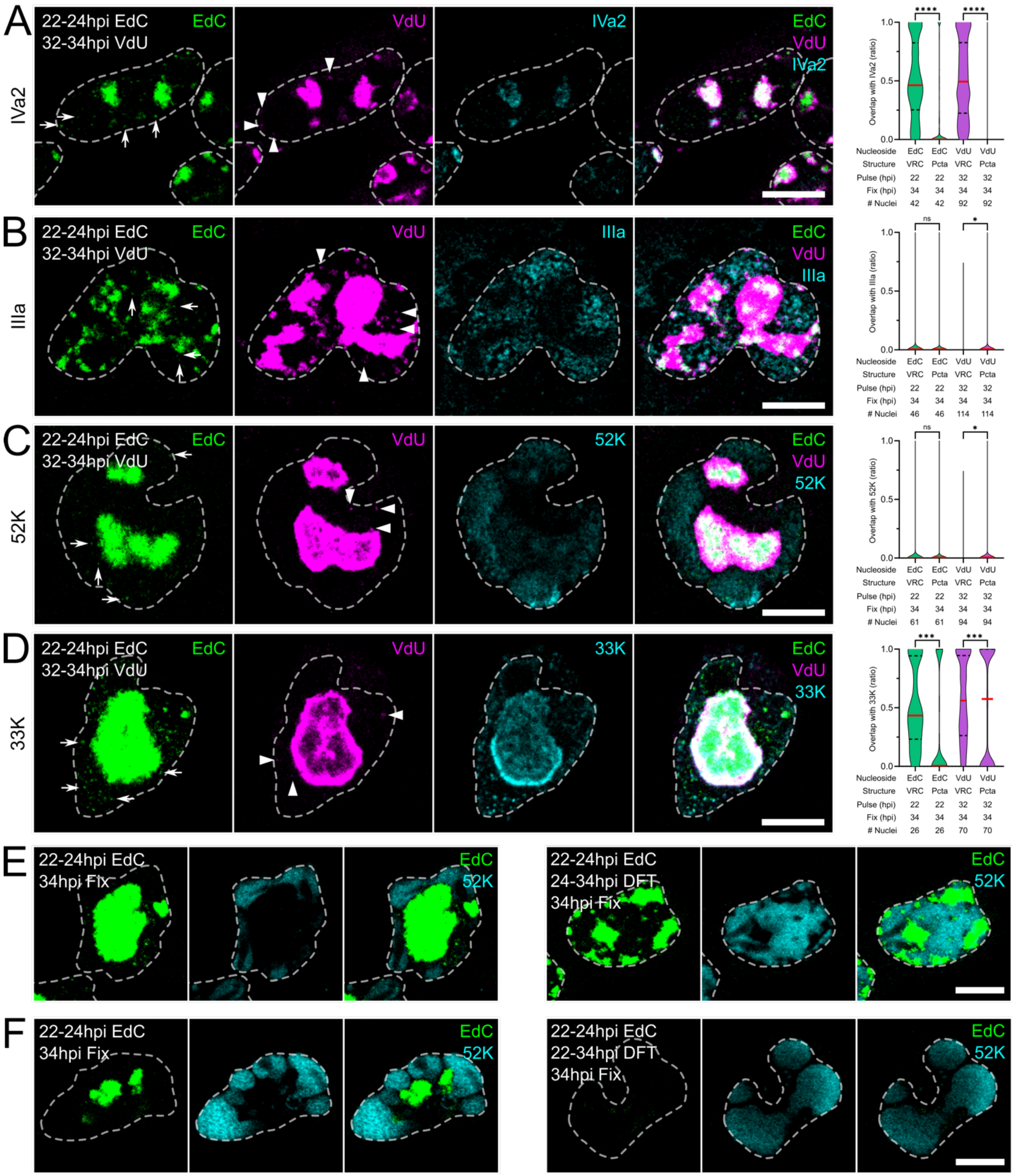

Figure S5: VRC but not nascent vDNA puncta colocalize with viral packaging proteins at late stages of infection (related to Fig. 4)

(A-D) Analyses of nascent vDNA puncta for vDNA packaging proteins IVa2, IIIa, 52K and 33K 34 hpi. Samples were prepared as described in Fig. 3C. VdU represents the VRC.

Data distribution is shown as violin plots. Puncta are indicated as Pcta. Median indicated in red. Statistical significance was determined by non-parametric ANOVA with Holm-Sidak for multiple comparisons. *, $p<0.03$; **, $p<0.0021$; ***, $p<0.0002$; ****, $p<0.0001$; ns, non-significant. Arrows indicate single EdC-puncta (labeled at 22 hpi); arrowheads indicate single VdU-puncta (labeled at 32 hpi). Representative images are single confocal slices. Scale bar, 10 µm.

(E) Inhibition of vDNA replication leads to VRC fragmentation but does not abrogate the 52K gel-like substance. Specimens were prepared as described in Fig. 3C. Cells were treated with 2.5 µM DFT at 24 hpi, and clicked with N_3-AlexaFluor594. Images are single confocal slices. Scale bar, 10 µm.

(F) DFT blocks the incorporation of EdC into the VRC. Specimen were prepared as described in Fig. 3C. Cells were treated with 2.5 µM DFT at 22 hpi, and clicked with N_3-AlexaFluor594. Images are single confocal slices. Scale bar, 10 µm.

Figure S6

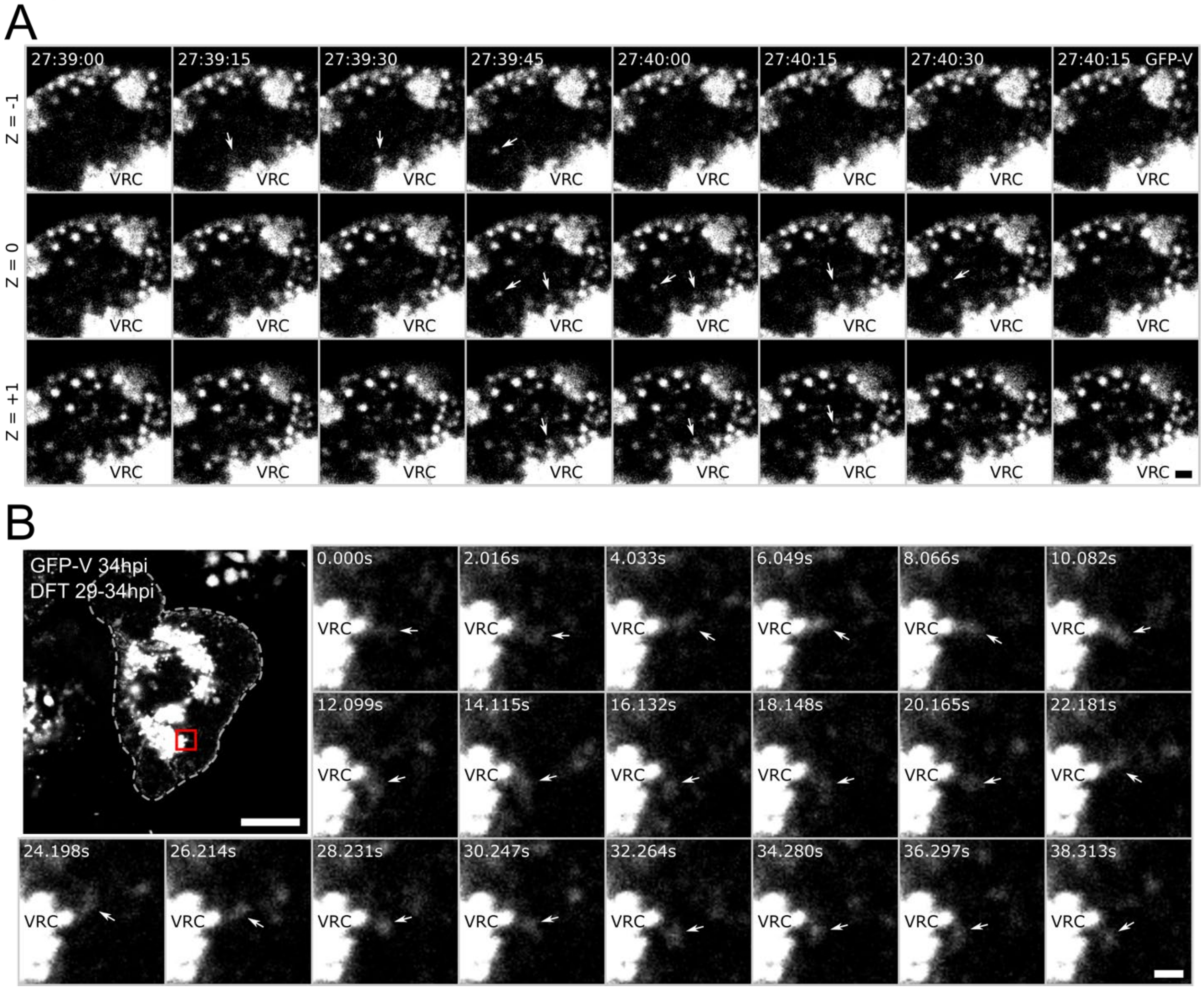

Figure S6: Bubbling of nascent GFV-V puncta from the VRC is replication-dependent (related to Fig. 5)

(A) Nascent GFP-V puncta bubble from the replication compartment, diffuse in the nucleus and eventually enrich in the nuclear periphery. A549 cells were infected with AdV-C2-GFP-V as described in Fig. 5C. GFP-V signal was recorded on whole cell Z-stacks from 27:30-28:00 hpi at a frequency of 0.07 Hz (4 full frames / min). Arrows indicate nascent GFP-V puncta. Images represent three contiguous Z planes, one in the middle (Z=0), one above (Z=1) and one below (Z=-1) the middle plane. Scale bar, 1 μm.

(B) GFP-V objects fail to dissociate from the VRC in presence of the replication inhibitor DFT. A549 cells were infected with AdV-C2-GFP-V as described in Fig. 5C. DFT was added at 29 hpi. GFP-V signal was recorded on a single Z-plane from 34:00-34:10 hpi at a frequency of 0.5 Hz in an Olympus IXplore SpinSR10 spinning-disk microscope. Arrows indicate a failed detachment attempt of a GFP-V object (grey color) from the VRC (white object). Frames are single confocal slices. Scale bar, 10 μm. Scale bar in the zoom-in, 1 μm.

Supplementary Movies

Movie S1: Cellular DNA clustering during AdV infection (10 fps)

A549 cells were grown in 25 µM VdU. Cells were infected with AdV-C5 (MOI 3) for 60 min, washed and labelled with 10 µM AO-6MT in FluoroBrite at 6-44 hpi. AO-6MT was recorded from 8-44 hpi in an IXMc confocal spinning-disk microscope at a frequency of four frames per hour. Timestamp h:min. The prominent extranuclear signals represent acidic compartments, where AO-6MT tends to accumulate over time. Frames represent maximum projections. Scale bar, 10 µm.

Movie S2: AdV-C5 VRCs visualized by AO-6MT (10 fps)

A549 cells were inoculated with AdV-C5 (MOI 3) for 60 min, washed and incubated with 25 µM VdU from 18-22 hpi, followed by 10 µM AO-6MT in FluoroBrite containing Hoechst DNA dye. AO-6MT was recorded from 23-65 hpi at a frequency of two frames per hour in an IXMc confocal spinning-disk microscope. Timestamp h:min. Frames represent maximum projections. Scale bar, 10 µm.

Movie S3: GFP-V puncta emerge from the VRC during late stages of AdV infection (5 fps)

A549 cells were infected with AdV-C2-GFP-V for 60 min, washed, pulse-labeled with 2.5 µM EdC at 22-24 hpi. GFP-V was recorded on full cell Z-stacks from 27:30-28:00 hpi in an Olympus IXplore SpinSR10 spinning-disk microscope at a frequency of four stacks per minute. Timestamp h:min:s. Frames are single Z slices. Scale bar, 10 µm.

Movie S4: Zoomed-in view of a GFP-V puncta formation at the periphery of the VRC (2 fps)

A549 cells were infected and imaged as described in movie S4. Timestamp h:min:s. Frames are single Z slices. Scale bar, 1 µm.

Movie S5: Nascent GFP-V puncta with restricted diffusion and unrestricted GFP-V positive AdV particles at late stages of infection (5 fps)

A549 cells were infected with AdV-C2-GFP-V as described movie S3. GFP-V signal was recorded on single confocal slices from 33:30-33:40 hpi in an Olympus IXplore SpinSR10

spinning-disk microscope at acquisition frequency of 0.5 Hz. Timestamp h:min:s. Frames are single confocal slices. Scale bar, 10 μm.

Movie S6: Zoomed-in view of nascent GFP-V puncta bubbling from the VRC at late stages of AdV infection (5 fps)

A549 cells were infected and imaged as described in movie S5. Frames are single confocal slices. Scale bar, 1 μm.

Movie S7: Failed dissociation of GFP-V objects in AdV infected cells treated with the replication inhibitor DFT (5 fps)

A549 cells were infected with AdV-C2-GFP-V as described movie S3. DFT (2.5 μM) was added at 29 hpi and kept for the rest of the infection. GFP-V signal was recorded on single confocal slices from 34:00-34:10 hpi in an Olympus IXplore SpinSR10 spinning-disk microscope at acquisition frequency of 0.5 Hz. Timestamp h:min:s. Frames are single confocal slices. Scale bar, 10 μm.

Movie S8: Zoomed-in view showing failed dissociation of GFP-V objects in AdV infected cells treated with the replication inhibitor DFT (5 fps)

A549 cells were infected and imaged as described in movie S7. Frames are single confocal slices. Scale bar, 1 μm.

Movie S9: Slow movement of nascent GFP-V puncta at the nuclear periphery during late stages of AdV infection (20 fps)

A549 cells were infected with AdV-C2-GFP-V as described movie S3. GFP-V signal was recorded on single confocal slices using an Olympus IXplore SpinSR10 spinning-disk microscope at acquisition frequency of 31.2 Hz. Timestamp ms. Frames are single confocal slices. Scale bar, 10 μm.

Movie S10: GFP-V positive viral particles diffuse with Brownian motion through the nucleoplasm during late stages of infection (20 fps)

A549 cells were infected with AdV-C2-GFP-V as described in movie S3. GFP-V signal was recorded in single confocal slices using an Olympus IXplore SpinSR10 spinning-disk

microscope at acquisition frequency of 31.2 Hz. Timestamp ms. Frames are single confocal slices. Scale bar, 10 μm.
